# Social identity and cooperation co-evolve in a multilevel public goods game

**DOI:** 10.1038/s41598-025-30979-2

**Published:** 2025-12-28

**Authors:** Charlie Pilgrim, Alexander J. Stewart, Nichola J. Raihani

**Affiliations:** 1 School of Mathematics, University of Leeds, Woodhouse, Leeds, LS2 9JT UK; 2 Dept of Experimental Psychology, University College London, 26 Bedford Way, London, WC1H 0AP UK; 3 Luddy School of Informatics, Computing, and Engineering, Indiana University Bloomington, Bloomington, IN 47408 USA; 4 School of Psychology, University of Auckland, 23 Symonds Street, Auckland, 1010 New Zealand

**Keywords:** Evolution, Psychology, Psychology

## Abstract

In the real world people often face collective action problems at multiple scales, where they must choose between local and global cooperation. Unlike single public goods scenarios, which can be solved if enough players are willing to cooperate, multilevel collective action problems introduce an additional coordination challenge in working towards the same goals. Using a pre-registered, online behavioural experiment, we investigate how perceptions of social identity predict and covary with cooperation success in multilevel public goods games. We find that introducing a local cooperation option undermines global cooperation and exacerbates parochial biases, but that increasing the payoffs of global cooperation boosts global cooperation levels. We also see a performance-cohesion effect, whereby successful cooperation within groups is associated with increased social identification with that group. Overall, our findings point to the importance of social context for cooperation, social identity, and their interplay.

**Supplementary Information:**

The online version contains supplementary material available at 10.1038/s41598-025-30979-2.

## Introduction

Collective action problems arise at many scales, from small-scale local dilemmas to large-scale global challenges. Although local investment is often important, action at the global scale is required to solve many of the most pressing problems we currently face^1–4^. Understanding the psychological mechanisms that promote or undermine global cooperation is therefore of real-world importance. How people behave when faced with a collective action problem can be studied under laboratory settings using public goods games^5–7^, which yield insights into the social preferences and strategic decision-making that underpin successful cooperation^8–10^. Nonetheless, previous work has typically focussed on how people behave when faced with singular public goods despite the fact that, in the real world, we belong to many social groups and can invest at many scales^11^. For example, with climate change^3,12^ or antibiotic resistance^2,13,14^, people can take individual actions (e.g. improving home energy efficiency, reducing antibiotic use), contribute to community-level initiatives (climate adaptation, local infection control), or support systemic policy at national and global levels (climate change mitigation, global antibiotic restrictions). The mismatch between experimental research and real-world complexity limits our capacity to understand how people choose between competing scales of cooperation.

A single public goods game contains a tension between individual self-interest and collective cooperation^6,7,15^. Individuals within a group receive an endowment and can choose to invest this in a communal pot (‘cooperate’) or to keep it for themselves (‘defect’/‘free-ride’). Canonically, contributions to the pot benefit the group through either a linear payoff rule, where the total pot is multiplied by a factor and shared equally throughout the group^16,17^ or a threshold payoff rule, where exceeding a threshold of investment yields a bonus for the entire group^8,10^. While total payoffs are higher when players cooperate, individuals are incentivised to free-ride (‘defect’) by letting others contribute and keeping their own endowment.

The multilevel setting introduces a more complicated structure in which each individual belongs to both a larger *global* group and a smaller *local* subgroup, and can choose to invest in the global pot, invest in their own subgroup’s local pot, or to defect by keeping their endowment^18–20^. Compared to a single public goods game, the multilevel game contains an additional tension: individuals must decide not only whether to cooperate but also at which scale. This introduces a coordination problem, since those who are willing to invest must still decide *which* public good to invest in^18,20–27^. The coordination problem is particularly acute in threshold multilevel public goods games. In threshold games the highest paying option depends on what others do: investing is optimal if that contribution is pivotal for reaching the threshold, but free-riding pays more if there are too few investments to reach the target or the number of investors exceeds that required to meet the target^8,10^.

Although threshold public goods games are arguably more reflective of real-world collective action problems^28–30^, they have been relatively understudied in experimental settings. Existing work has found that multiple investment options result in lower contributions and reduced group success in meeting thresholds^24–26,31^. However, successful coordination can be increased by the introduction of a salient focal point for investment^24^ or an intermediary to coordinate investments^25,31^.

Studies exploring investment strategies in multilevel public goods games have often found that people prefer to invest locally^11,32,33^. This parochialism can arise for a number of reasons. People may experience a greater sense of social identity with their local group^34–37^ and invest accordingly (e.g.^27^ ). People may also prefer to invest locally rather than globally because the local option may be perceived as easier to meet, as it typically requires fewer people to coordinate to meet a local target^38,39^. While parochialism is common, it is not universally observed. For example, people from countries with a higher globalization score, or who report a higher sense of identifying with the world community, are more likely to invest in a global rather than a local public good^21,22^. Moreover, parochial biases can be mitigated and global cooperation can be effectively incentivised by increasing the payoffs for global-scale cooperation^18,19,23,27,40^.

While cooperation is also influenced by perceptions of social identity^21,22,35,37^, other work points to a reciprocal effect, whereby successful groups become more cohesive^41–43^. This raises intriguing questions of how cooperation and social identity interact over time, how that interaction is influenced by the social context, and how this cultural behavioural dynamic evolves within and across groups.

Here we present an online experiment comprising a repeated, multilevel threshold public goods game designed to address two main research questions: (1) does the presence of a local option for cooperation undermine cooperation at a higher level; (2) do increased incentives at the global level overcome any reduction in global cooperation resulting from the presence of a local option. In addition to individual behavior, we explore how perceptions of social identity shape^21,22,35^—and are shaped by ^41–43^—patterns of cooperation in the game. The multilevel structure allows us to alter the social context through experimental conditions that vary the opportunities for cooperation at different scales. The social context is important: we find that the presence of options for local cooperation undermines global cooperation, which can be recovered through greater incentives at the global level. Furthermore, social identity plays a key role: both influencing, and being influenced by, levels of cooperation in a co-evolving dynamic.

Methods.

This project was approved by the UCL Ethics Committee (project number 3720-002). All methods were performed in accordance with the relevant guidelines and regulations. Participation was voluntary and all participants provided informed consent prior to study participation. We recruited 1401 English-speaking participants from the online recruitment platform, Prolific (https://www.prolific.com/), to play a multilevel public goods game involving cooperation at local and global scales (described below). A total of 1197 participants completed the game (594 identifying as female, 602 identifying as male, 1 unknown).

**Fig. 1:**
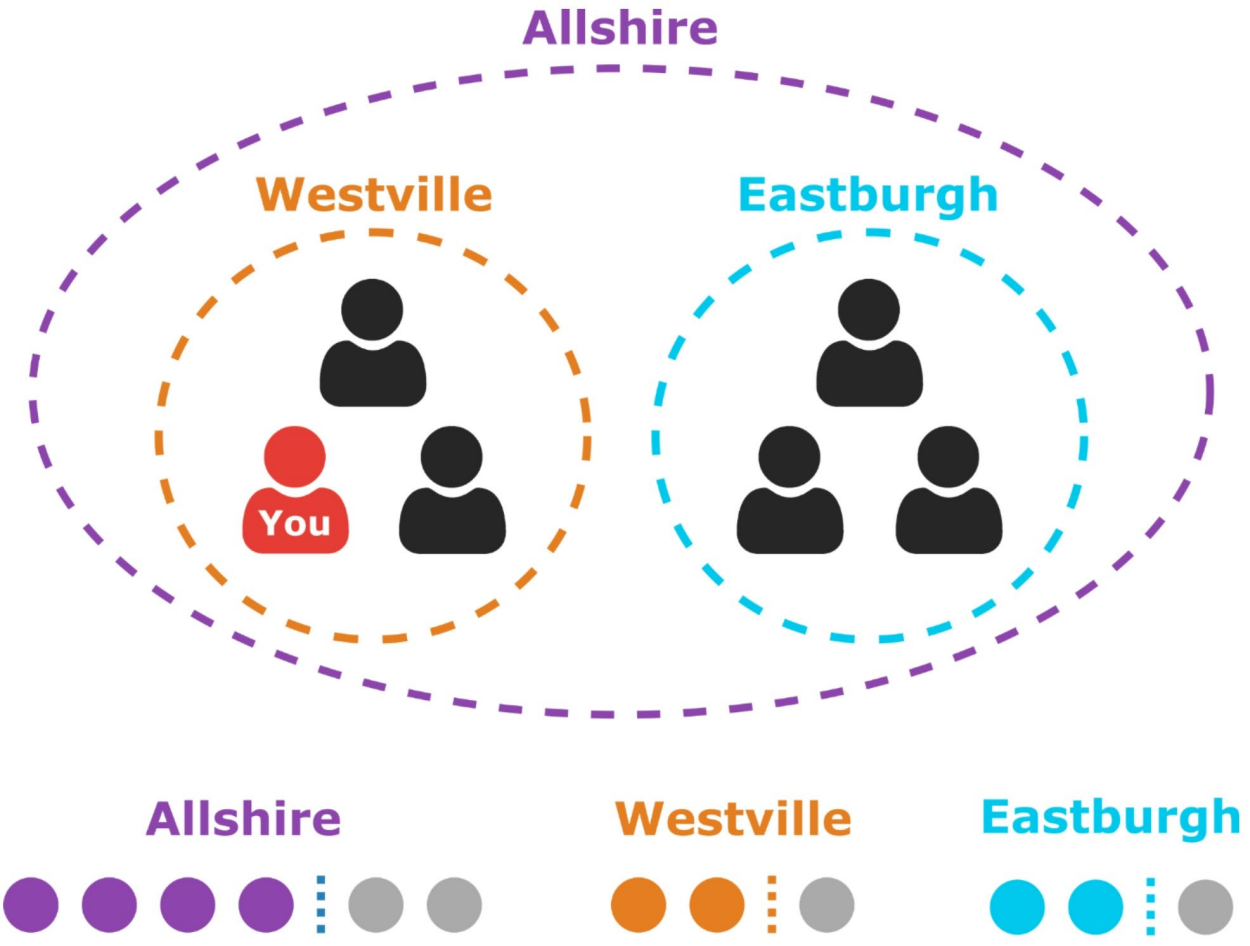
The group structure of the game as described to the participants. Participants play in a global group of 6 (Allshire) as well as in their own local group of 3 (Westville). Each round involves simultaneous threshold public goods games played at both the global and local level. The threshold for the global group is 4 investors, while the threshold for each local group is 2 investors. The Global Only condition has the same group structure but participants do not have the option to invest in the local groups.

The study design was pre-registered (https://osf.io/me9j3/?view_only=c6ea2eee3e5d4c50b57725db2969edb8) and all analyses follow the pre-registration unless stated otherwise. Participants were recruited to play a web-based multilevel threshold public goods game. Each participant was told that they were a member of a global group (‘Allshire’) of six players, and a member of their own local group (‘Westville’) of three players. Participants were told that the ‘other’ local group was called ‘Eastburgh’ (Fig. 1). To maintain consistency, every participant received identical instructions, labelling their own subgroup as Westville and the other subgroup as Eastburgh. In the experimental software, groups were split into two subgroups, and choices to invest in Westville were internally coded as investments in the participant’s own local subgroup, while choices to invest in Eastburgh were internally coded as investments in the other local subgroup.

Participants played an iterated game over 20 rounds with the same group. During the instructions, players were told that they would play with the same players. To minimise the effect of end-game changes in strategy^44^, players were told that they would play up to 25 rounds, although the game concluded after 20 rounds.

In each round participants received a single experimental coin which they could choose to invest or keep for themselves. If participants chose to invest their coin, they could contribute to their own local group (Westville), to the other local group (Eastburgh), or to the global group (Allshire). Participant contributions to the other local group were counted towards the other subgroup’s total invested coins. Despite providing no potential payoff to the participant, the option to invest in the other local subgroup was included to make it clear that local contributions were tracked separately for each subgroup. Such choices were extremely rare (0.6%).

At the end of each round participants were told the total number of coins invested in their own local group (Westville), the other local group (Eastburgh), the global group (Allshire), and the number of coins kept. They were also informed which thresholds had been met and their total payoff for the round.

Each participant played in one of three experimental conditions (Table 1). In the Balanced and Global Boost conditions, participants received payoffs from their own local group and from the global group if the respective payoff thresholds were met. In the Global Only condition, there was no option to invest locally and no local payoff available. In all three conditions participants could choose to keep the coin rather than invest it. Thus, in all three conditions participants received payoffs from successfully meeting cooperation thresholds, but could potentially free-ride on others’ contributions by keeping their own coin rather than investing it.

**Table 1:** Payoffs for meeting thresholds in the three experimental conditions from the perspective of a focal participant in Westville. The payoffs were given each round as an in-game currency of “coins”. The coins were converted to bonus payments at a rate of 1 coin = £0.05. The global only condition had no option to invest in local groups and no local payoff available. Participants could receive multiple payoffs each round if the conditions were met.

Payoff condition	Who gets the payoff	Payoffs in conditions		
		Global only	Balanced	Global boost
Global threshold met (≥ 4 invested in Allshire)	All members of Allshire	+ 2	+ 2	+ 2.5
Own local threshold met (≥ 2 invested in Westville)	All members of Westville	N/A	+ 2	+ 2
Other local threshold met (≥ 2 invested in Eastburgh)	All members of Eastburgh	N/A	+ 2	+ 2
Kept coin	Participant who kept their coin	+ 1	+ 1	+ 1

Our primary research question was whether the presence of a local option for cooperation would undermine cooperation at a global level. To answer this question we minimally needed two conditions: the baseline Global Only condition without local cooperation, and the comparison Balanced condition with local cooperation. Our secondary research question was whether increasing global incentives would recover global cooperation. Hence, we included the Global Boost condition where the payoff for meeting the global threshold exceeded that for meeting the local threshold (Table 1).

We characterised the Nash equilibria for each condition to clarify the strategic structure of the game^45^. We summarised action profiles as (G, L_1_, L_2_, D) where G was the number of global contributors, L_1_ and L_2_ were the number of investors in each local subgroup, and D was the number of defectors. Note that G + L_1_ + L_2_ + D = 6. Although mixed strategy Nash equilibria exist, we focused on the pure equilibria, which more intuitively describe the coordination structure of the game. The pure Nash equilibria in the Global Only condition were (4,0,0,2) and (0,0,0,6). The pure Nash equilibria in both the Global Boost and Balanced conditions were (4,0,0,2), (4,2,0,0), (4,0,2,0), (0,2,2,2), (0,2,0,4), (0,0,2,4), (0,0,0,6). In short, Nash equilibria consisted of combinations of minimal winning coalitions at the global and/or local levels, with full defection also constituting an equilibrium^10,45^.

A full description of comprehension checks and pre-registered exclusion criteria are available in the Supplementary Information. We collected data for *N* = 171 full groups of 6 players, split equally between the 3 conditions yielding 57 groups per condition. Participants were paid £2.50 for taking part, and earned further bonuses averaging £1.81, equivalent to 36.2 in-game coins (Global Only = 34.6 coins; Balanced = 32.4 coins; Global Boost = 41.6 coins).

### Perceptions of social identity

We used the Dynamic Identity Fusion Scale (^46^MIT open license https://github.com/Dallinger/identityfusion?tab=MIT-1-ov-file) to measure participants’ perceptions of social identity with their own local, other local and global groups. These measurements were taken prior to the game (round 0) and subsequently after rounds 5,10,15, and 20. The Dynamic Identity Fusion Scale is designed to measure the extent of “oneness” with a group, and is a dynamic version of the pictorial identity fusion scale^47^ that aims to measure the overlap in personal and social identities. Accordingly, we interpreted these variables as indicators of each participant’s perceptions of social identity in relation to each group.

To obtain social identity estimates, participants were instructed to drag a circle labelled “Me” to another circle representing each of the global, own local and other local groups. Participants read the following instructions: “The diagram below is designed to represent your relationship with Allshire (/Westville/Eastburgh). Please indicate your relationship by clicking and dragging the smaller ‘Me’ circle to the position that best captures your relationship with Allshire (/Westville/Eastburgh)”. The final x-position of the “Me” circle was transformed onto a 0–100 scale to yield three variables for perceptions of identity with participant’s own local, other local and the global group.

### Pre-registered analysis

The pre-registered analysis plan (https://osf.io/me9j3/?view_only=c6ea2eee3e5d4c50b57725db2969edb8) is summarised in Table 2. Full details are in the Supplementary Information.

Our main research questions were (1) is global-scale cooperation undermined by the option to invest at local scales?; and (2) can increasing incentives at the global scale recover global cooperation? The analyses were consistent with a norm-utility theoretical framework^48^, whereby individuals make decisions that balance material payoffs and adherence to social norms, with competing normative expectations in multilevel public goods. Existing research finds that competing options for cooperation reduces cooperation success rates^24–26,31^, with local cooperation preferred^11,32,33^. Cooperation can be recovered by increasing incentives^8^ and saliency^24^, which we expect can serve as a coordination device through increasing expected payoffs of cooperation and reducing normative uncertainty. We also expected to replicate trends in declining cooperation over time^49,50^ as failures to coordinate reinforce norms of non-cooperation.

Drawing on existing experimental and theoretical work, we expected perceptions of social identity^35,36^ to predict cooperation decisions in multilevel public goods games, consistent with perspectives on social identity as a heuristic for managing complexity and uncertainty in social environments^37,51,52^. Existing work suggests that perceptions of social identity will vary between groups^11,21,32,33,53–55^ and be associated with cooperative behaviour^20–23,27^. Reciprocally, group success can also improve perceptions of social identity in the performance-cohesion effect^41–43^.

**Table 2:** Pre-registered analysis plan.

Analysis	Prediction	Analysis approach
H1a	Adding a local option will reduce global cooperation	Mann–Whitney U test comparing global cooperation rates between Global Only and Balanced conditions
H1b	Adding a local option will increase total cooperation	Mann–Whitney U test comparing defection rates between Global Only and Balanced conditions
H2a/b	Increasing global payoffs will increase (a) global and (b) total cooperation	Mann–Whitney U tests comparing (a) global cooperation and (b) defection rates between Global Boost and Balanced conditions
H3a/b	Over the course of the game there will be a decline in (a) global and (b) total cooperation	Cumulative link mixed models (CLMMs) with round as predictor for (a) global cooperation and (b) defection rates
H3c	No directional prediction for local cooperation trends. *Note that this is not technically a hypothesis, it is a pre-registered analysis	CLMM with round as predictor for local cooperation rates
H4a	Initial social identity will be stronger with participants’ own local than the other group	Wilcoxon signed-rank test for pairwise comparisons of initial identity perceptions between participants’ own local and the other group
H4b	Initial social identity will be different between participants’ own local and the global group	Wilcoxon signed-rank tests for pairwise comparisons of initial identity perceptions between participants’ own local and the global group
H5	Initial social identity will predict first-round actions	Logistic regression with global-own local identity difference as a predictor for global vs. own local first round cooperation
H6	Changes in social identity will be associated with cooperation success at the global and local level	Linear mixed effect regression models with number of thresholds met as a predictor for changes in identity
H7	Social identity will decline over the game	Linear mixed effects regression models with round as predictor for identity

## Results

**Fig. 2:**
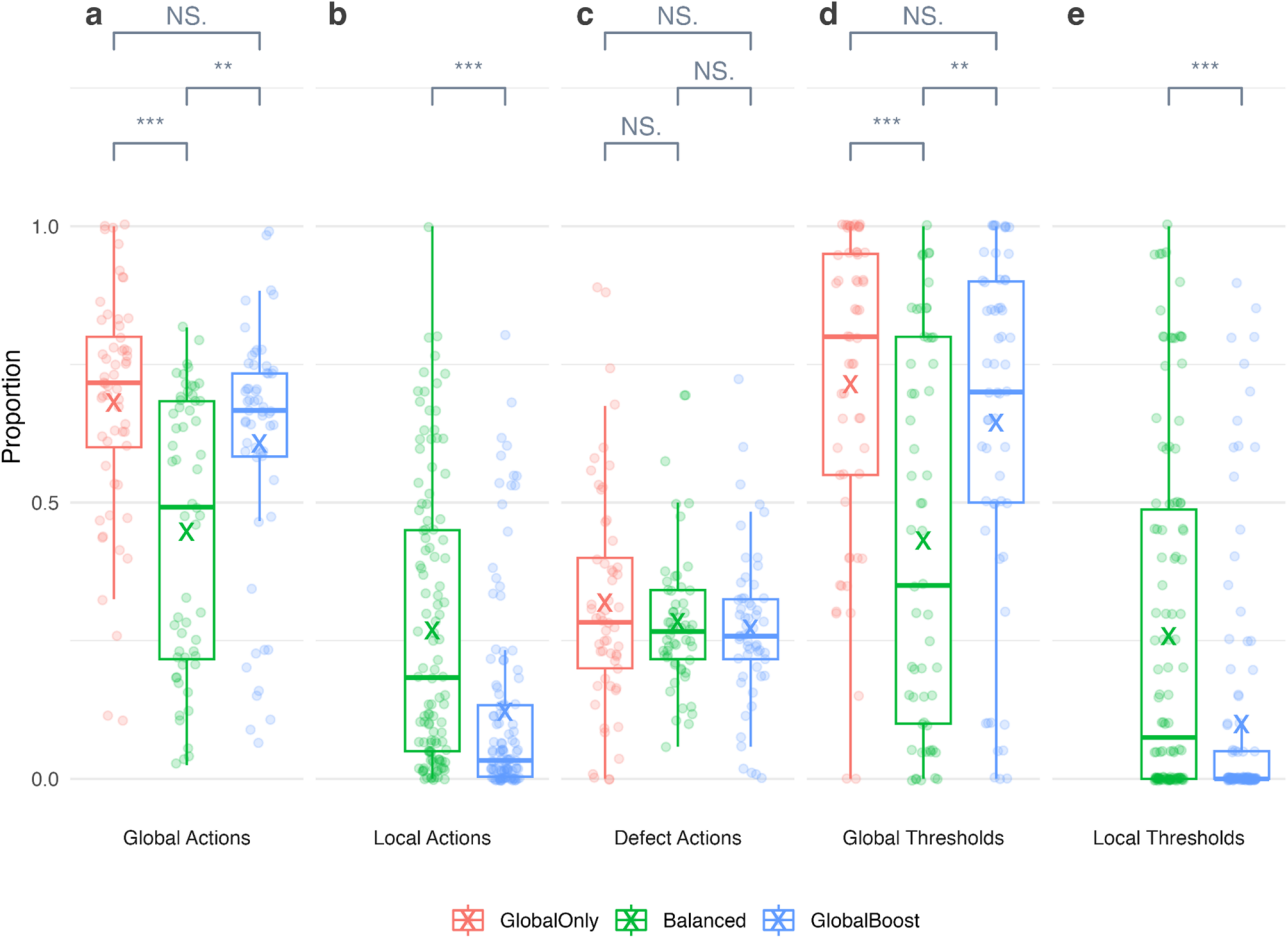
Proportions of actions (**a**–**c**) and thresholds met (**d**,**e**) in groups across the experimental conditions. Boxplots show distributions, with whiskers to 1.5 times the interquartile range. Each jittered circular point shows the proportion for a single group and crosses denote mean rates. Significance levels, adjusted using a Holm–Bonferroni correction for multiple comparisons, are denoted as follows: NS *p* > 0.05, *p < 0.05, **p < 0.01, ***p < 0.001.

Across all experimental conditions, participants chose to invest globally in 57.7% of rounds and to defect in 29.0% of rounds. When the local option was available, participants chose to invest in their own local subgroup in 18.8% of rounds and the other local subgroup in just 0.6% of rounds. This behaviour translated to global thresholds being met in 59.7% of rounds. Where local investment was possible, local thresholds were met in 17.9% of rounds (Fig. 2).

### Cooperation

Adding a local option reduced global cooperation (H1a supported). Global investments were lower in the Balanced (44.7%) compared to the Global Only (68.1%) condition (W = 2490, *p* < 0.001; Fig. 2a), and groups were less likely to meet the global threshold targets in the Balanced (43.2%) than the Global Only (71.4%) condition (W = 2410, *p* < 0.001; Fig. 2d).

Nevertheless, the addition of a local option did not affect rates of total cooperation (H1b unsupported). Defection rates were comparable in the Balanced (28.4%) and Global Only (31.9%) conditions (W = 1770, *p* = 0.85; Fig. 2c). The reduction in global cooperation in the Balanced vs. Global Only condition was offset by increased local cooperation in the Balanced condition (Fig. 2b).

As predicted, increasing global payoffs increased global cooperation (H2a supported). Global investments were significantly higher in the Global Boost (60.7%) compared to the Balanced (44.7%) condition (W = 1052, *p* = 0.008; Fig. 2a), and global thresholds were accordingly more likely to be met (W = 1068, *p* = 0.009; Fig. 2d).

Increasing global payoffs did not increase total cooperation (H2b unsupported). Defection rates were similar in the Global Boost (27.1%) and Balanced (28.4%) conditions (W = 1645, *p* = 0.91; Fig. 2c). Instead, increased global cooperation in the Global Boost condition was accompanied by lower local cooperation (12.1%) compared to the Balanced (26.9%) condition (W = 9140, *p* < 0.001; Fig. 2b). Accordingly, local thresholds were met less often in the Global Boost (10.0%) compared to the Balanced (25.8%) condition (W = 8660, *p* < 0.001; Fig. 2e).

**Fig. 3:**
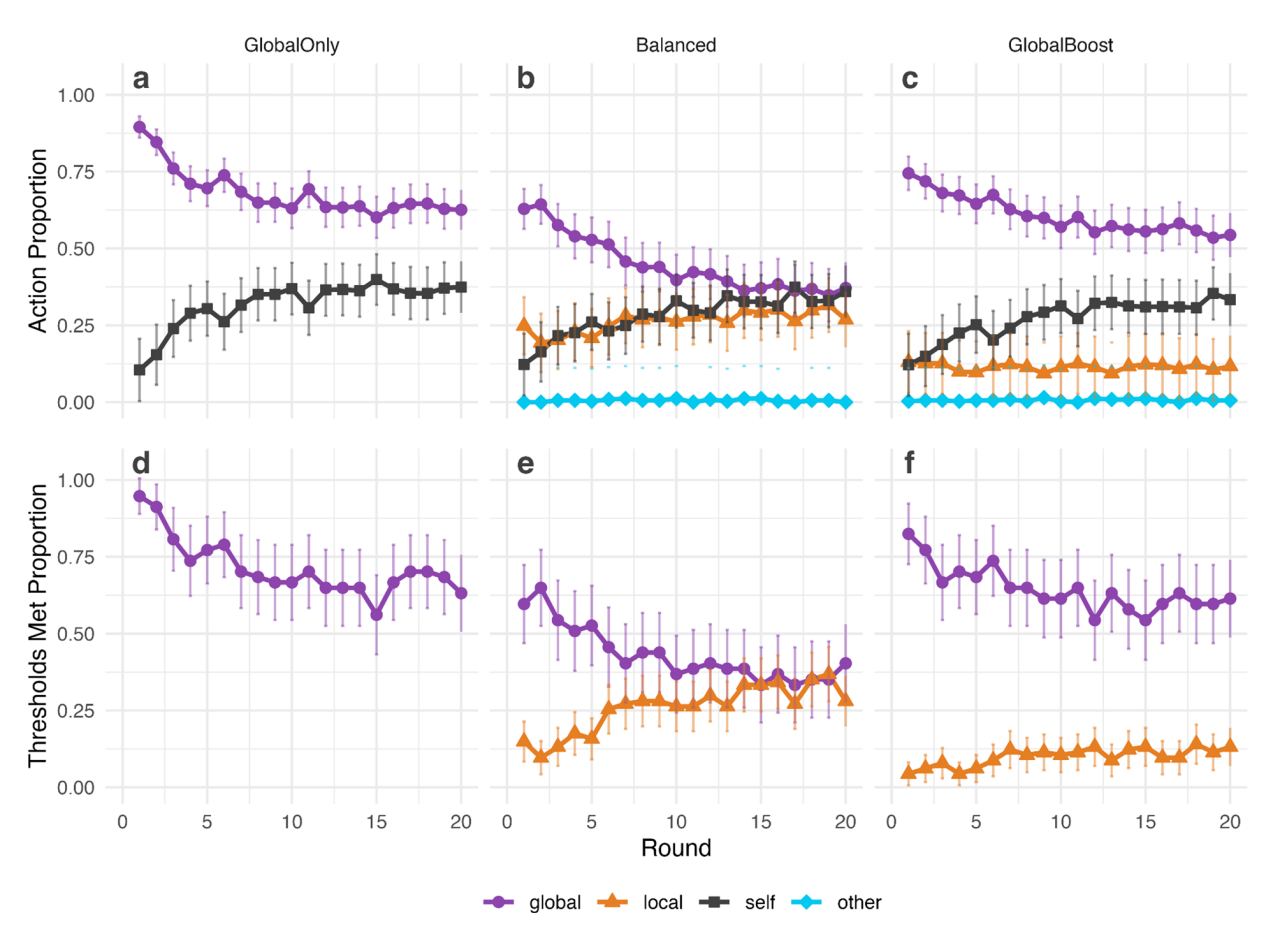
Action proportions (**a**–**c**) and thresholds met (**d**–**f**) over rounds in each experimental condition (*N* = 57 groups per condition). (**a**–**c**) Global cooperation declined and defection rose in all conditions. Local cooperation increased over rounds in the (**b**) Balanced condition but slightly decreased in the (**c**) Global Boost condition. (**d**–**f**) Global threshold success rates declined in all conditions. Local threshold success rates increased in the (**e**) Balanced condition and (**f**) Global Boost condition. Error bars show confidence intervals in mean proportions. See Supplementary Information Figs. S3 and S4 for more detailed analysis of the action counts across groups, which shows some bimodality.

Defection rose over the course of the game across all conditions (H3b supported; Fig. 3; CLMM coefficient = 0.12, *p* < 0.001). Global cooperation decreased as the game progressed in all conditions (H3a supported; Fig. 3; CLMM coefficient = -0.14, *p* < 0.001) but local cooperation trends varied across conditions (Fig. 3, H3c).

Local cooperation increased over rounds in the Balanced condition (Fig. 3b; CLMM coefficient = 0.03, *p* < 0.001). A linear combination of regression estimates provided evidence of a downward trend in local cooperation in the Global Boost condition (local model combined *round* and round*Global Boost coefficient = -0.02, *p* = 0.02). This small effect is obscured by between-group variance in Fig. 3 but is more clearly visible when data are centered by group (Fig. S1).

Full statistical results are shown in the Supplementary Information. The CLMM model, as well as showing the trends reported here, also reaffirms the results comparing overall levels of cooperation across conditions (Table S2).

### Social identity

Participants reported higher initial perceptions of social identity with their own local group than the other local group, as expected (H4a supported; Wilcoxon signed rank tests for each condition, all *p* < 0.001; Table 3). Similarly, participants identified more with their own local group than the global group in each condition, as predicted, though these differences were small (H4b supported, Wilcoxon signed rank tests, all *p* < 0.01; Table 3). In an exploratory analysis, we found that the addition of a local option for cooperation (in the Balanced and Global Boost conditions) was associated with lower initial perceptions of social identity with the other local group than in the Global Only condition (all Wilcoxon signed rank tests *p* < 0.001; Table 3).

**Table 3:** Mean initial perceptions of social identity ± standard deviation (standard error), for local and global groups across conditions.

	Global Only	Balanced	Global Boost
Own local	91.9 ± 12.6 (0.83)	93.4 ± 11.6 (0.67)	93.2 ± 10.9 (0.64)
Other local	62.6 ± 23.4 (1.55)	47.2 ± 27.3 (1.57)	50.1 ± 27.0 (1.60)
Global	90.5 ± 12.1 (0.87)	89.9 ± 11.5 (0.66)	90.7 ± 11.9 (0.70)

**Fig. 4:**
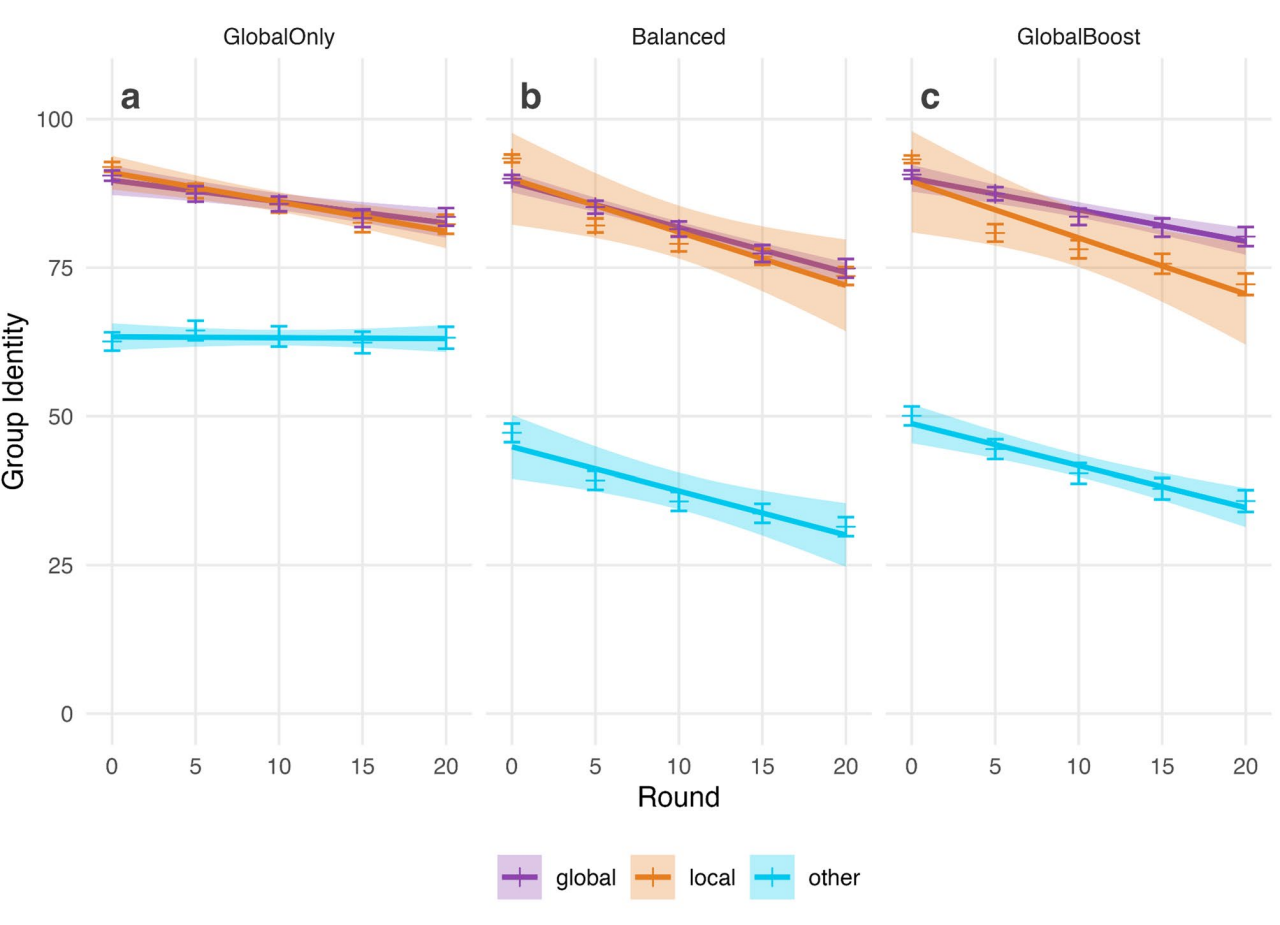
Trends in social group identity. Perceptions of identity with global and own local groups decreased over the course of the experiment. (**a**) In the Global Only condition, perceptions of identity with the global and own local group declined, while identity with the other local group remained constant. (**b**) In the Balanced condition, identity declined at a similar rate in all groups. (**c**) In the Global Boost condition, perceptions of identity with the global group fell more slowly than perceptions with the own local group. Points show the mean ratings of identity across individuals with error bars showing the standard error of the mean. Line fits are linear regression models with shaded regions showing 95% confidence intervals.

Perceptions of initial social identity predicted first round actions (H5 supported). Across all conditions participants were more likely to invest in the global vs. own local group in the first round if they reported higher initial social identity with the global group than their own local group (logistic regression *global minus local identity* coefficient = 0.03, *p* = 0.04).

Subsequent perceptions of social identity were associated with cooperation success. In general, perceptions of social identity with all groups declined over the game (H7 supported; Fig. 4; global model *round* coefficient = − 0.76, *p* < 0.001; own local model *round* coefficient = − 0.90, *p* < 0.001; other local model *round* coefficient = − 0.74, *p* < 0.001), reflecting the decreased tendency to meet the cooperation thresholds as the game progressed. Nevertheless, we also observed a performance-cohesion effect (H6 supported), whereby local/global cooperation success was associated with increased perceptions of social identity at that scale (global perception model success coefficient 2.09, *p* < 0.001; own local perception model success coefficient 1.35, *p* < 0.001). Full statistical results are in the Supplementary Information (Table S6; Fig. S2).

## Discussion

Our study was motivated by two main research questions: whether the presence of a local option for cooperation would undermine global cooperation, and whether global cooperation would be recovered through increasing incentives. As predicted, and consistent with previous work, we found that adding a local option reduced global contributions^24–26,31^. We expected this effect not only because local cooperation is easier, but also because the introduction of an additional option means that there is no clear Schelling focal point^56^, making coordination more difficult. Global cooperation was restored when global incentives were increased, as expected. This effect is consistent with two connected hypotheses: higher payoffs made global cooperation more attractive and could have acted as a salient coordination device^8,24,57^. We expected the introduction of a local option to increase total cooperation levels, but this prediction was unsupported. Nor did increasing global payoffs increase total cooperation. Instead, cooperation shifted between local and global levels. Together, these results indicate that the overall social context—in this case, the presence of local alternatives and the relative size of payoffs—influence decision making and group outcomes in collective action problems.

Real-world collective action problems often play out in complicated social contexts with opportunities for action at multiple scales^1–4^. In climate change, local adaptation measures (e.g. flood defenses) may undermine investment in global mitigation efforts^3,12^. In antibiotic resistance, local infection control reduces individual antibiotic use, but global cooperation is required to develop new drugs and prevent resistant strains^2,13,14^. Our results highlight that opportunities for action at one scale can influence behaviour at others, an important consideration when structuring policy and incentives to tackle multiscale problems.

We were also interested in how perceptions of social identity were associated with decisions. As predicted, initial perceptions of social identity were associated with cooperation decisions in the first round of the game (cf.^20–23,27^). Furthermore, by tracking social identity across the game, we detected a predicted performance-cohesion effect^41–43^, whereby cooperation at the local or global level and social identity with those groups co-evolved. This co-evolution is consistent with social identity acting as a dynamic mechanism for conditional cooperation: while social identity provides a heuristic to manage reciprocity in complex social settings^37,51,52^, our findings show that social identity itself responds to group success, amplifying or dampening cooperation.

At the individual level, we observed conditional cooperation both locally and globally, as well as a tendency to repeat the last action taken (see pre-registered analysis in Table S14). Specifically, players increased their cooperation in response to higher proportions of cooperators in the previous round, though this relationship weakened at higher cooperation levels (with a negative quadratic term), suggesting players withdrew cooperation once sufficient others were contributing. These patterns are consistent with evidence for conditional cooperation in public goods games^58,59^ and behavioural strategies like tit-for-tat and win-stay-lose-shift^60,61^.

To better understand the behavioural dynamics connecting individual behaviour to collective outcomes, we carried out post-hoc exploratory analyses (detailed in Supplementary Information). Behavioural patterns stabilised in the later phase of the game (rounds 11–20), indicating that later game cooperation patterns represent relatively stable equilibria as opposed to transient dynamics. Most participants displayed consistent action tendencies in that they took the same action in over two thirds of rounds, while relatively few could be classified as strategic best-responders. Correspondingly, when groups achieved sustained cooperation this tended to rely on a stable core of contributors with only occasional switching, suggesting that coordination emerged through role differentiation of participants into action types.

In an exploratory analysis, we found that although the presence of local cooperation options did not affect perceptions of global identity, they did affect how people saw the other local group. Perceptions of identity with the other local group were initially lower, and declined more, in both the Balanced and Global Boost condition compared to the Global Only condition. One explanation is that local cooperation could compromise global investments in Balanced and Global Boost conditions (cf.^62^ ), meaning that members of the other local group were more likely to be viewed as competitors^35,63^, despite the fact that all players are members of the same global group. Perceptions of social identity did not always align with the functional relevance of the global, own local, and other local group. In the Global Only condition there were similar perceptions of social identity with the global and own local groups, but lower perceptions with the other local group, despite threshold rewards only being available at the global level. This pattern reflects the psychological saliency of ingroup membership whereby individuals prefer their own subgroup over other subgroups^11,63,64^, even within a global superordinate group structure^65,66^. This ingroup–outgroup asymmetry can arise rapidly even under arbitrary or random group assignments^67,68^ and is associated with preferences to allocate resources to the ingroup^69,70^.

We note a number of key limitations with this study. The multilevel public goods game is abstract and involves low stakes and small group sizes relative to the social dilemmas that we face in the real world. Such features compromise external validity (in that we should not expect the same magnitude of effects to be observed in real-world settings^71,72^. In particular, whereas each participant’s decision was relatively consequential for global cooperation success in our study, in many real-world global social dilemmas the pivotality of each person’s contribution is vanishingly small^38,39^. In part, the small group sizes (and associated thresholds for success) could explain why we observed relatively high levels of global cooperation in this experiment. Future work could explore cooperation in larger games^16,73^, though we note that people do contribute to large-scale, real-world cooperation where there is minimal chance of individual impact (e.g. elections^74,75^. People cooperate at a higher rate than predicted by purely rational theories^3,76,77^, and cooperation is predicted by a disparate and complex set of motivations^78,79^. We do not attempt to identify all of these here, but simply ask how changing a feature of an interaction (adding a local cooperation option) affects global cooperation, under the assumption that all else being equal the same direction of effects should be observed in real-world settings^71,80^. Importantly, because our study only involved Western participants, we cannot be sure whether we would observe the same direction of effects in other societies^81,82^.

In summary, our experiment expands research on multilevel threshold public goods games by introducing repeated behavioural interactions as well as tracking changes in social identity. We found that adding a local option for cooperation undermined global cooperation, but that global cooperation was recovered by increasing incentives. Total levels of cooperation did not differ significantly across conditions, but cooperation shifted between local and global scales. In addition, we observed a co-evolving dynamic between social identity and cooperation at the local and the global level: stronger identification with the own local or global group predicted more cooperation at that scale, while successful cooperation in turn strengthened social identity with the corresponding group. Together, our findings highlight the importance of the structural and social context for understanding cooperation in collective action problems.

## Supplementary Information

Below is the link to the electronic supplementary material.

Supplementary Material 1

## Data Availability

Data, analysis code, and code for running the experiments can be found at the repository: (https://github.com/chasmani/PUBLIC_social_identity_and_cooperation).
