# Supplementary material for: Social identity and cooperation co-evolve in a multilevel public goods game
Source: Sci Rep. 2025 Dec 28;16:1610. doi: 10.1038/s41598-025-30979-2 (PMC12800250; doi:10.1038/s41598-025-30979-2)
Supplement: Supplementary file 1 — Supplementary Material 1 [file 41598_2025_30979_MOESM1_ESM.pdf]

Supplementary Information. Social identity and cooperation co-evolve in a multilevel public goods game

Charlie Pilgrim*[a,b], Alexander J Stewart[c], Nichola J Raihani[b,d]

[a]School of Mathematics, University of Leeds, Woodhouse, Leeds LS2 9JT
[b]Dept of Experimental Psychology, University College London, 26 Bedford Way, London WC1H 0AP
[c]Luddy School of Informatics, Computing, and Engineering, Indiana University Bloomington, IN, 47408, USA
[d]School of Psychology, 23 Symonds Street,University of Auckland, Auckland 1010 New Zealand

*Corresponding Author: c.p.pilgrim@leeds.ac.uk

Supplementary Information. Social identity and cooperation co-evolve in a multilevel public goods game 1
1. Recruitment and Sample Size 1
2. Pre-Registered Hypotheses 3
3. Full Analysis Details 4
4. Results: Detailed Statistical Analyses 7
Comparisons of Actions and Thresholds Met Across Conditions 7
Trends of Actions During the Game 7
Comparison of Initial Perceptions of Social identity. 9
Trends in Perceptions of Social Identity 11
Performance Cohesion Effect 12
Predicting First Round Actions From Social Identity 14
5. Further Pre-registered Analyses 15
Mutual Dependence and Conflict 15
Hypotheses and Results 16
6. Further Exploratory Analyses 24
Action Distributions Timeseries 25
Group Dynamics 25
Intergroup Correlation Between Social Identity and Conflict Mutual Dependence 27
Behavioural Convergence 27
Behavioural Classification 29
Successful Strategies 30
References 31

1. Recruitment and Sample Size

This project was approved by the UCL Ethics Committee (project number 3720-002), all participation was voluntary and participants were informed as to the nature of the study prior to taking part. We recruited 1401 English-speaking participants from the online recruitment platform, Prolific (https://www.prolific.com/), to play a multilevel public goods game involving cooperation at local and global scales, which was developed using oTree [1].

A total of 1197 participants completed the game (594 identifying as female, 602 identifying as male, 1 unknown). The experiment was pre-registered (https://osf.io/me9j3/?view_only=c6ea2eee3e5d4c50b57725db2969edb8) with an initial study , to which we subsequently added two follow-up conditions. Sample sizes were determined by power analyses (through calculation and simulation) targeting a significance level of α=0.05 and a power of 0.8 for hypotheses. Full details, including simulation code, can be found in the pre-registration project files .

All exclusion criteria were detailed in the pre-registration. Before starting the game, participants were asked 13 comprehension checks over five pages, which also served as a tutorial for the game. Pages 1-3 contained instructions and questions about a) the number of rounds b) the group composition and c) the thresholds and bonus payouts. Pages 4-5 described hypothetical player actions and asked participants to determine the consequent bonus payouts for themselves and others. On each page, participants were unable to proceed until they answered all of the questions correctly. In the event of an incorrect answer, participants were informed that one or more of their answers were incorrect, but not which ones. This was done to prevent participants using trial and error to pass the comprehension checks. 135 out of 1401 participants left the experiment at this stage without completing the comprehension checks. A further 69 participants left the game itself without finishing, leaving a total of 1197 participants who finished the game.

We employed different exclusion criteria for the group and individual level analyses.

Group Level Exclusion Criteria

In each round, players were given 30 seconds to decide how to invest their coin. If they did not answer within 30 seconds, their decision was made by a bot. The actions of bots were generated by sampling from a probability distribution over the available actions, in proportion to the frequency of actions played in the group over all rounds so far. In the first round, this was replaced with a uniform probability distribution. If a player failed to answer in time in any three rounds then they were considered inactive and dropped out of the game, and the group data was excluded from the group level analyses (N=19 groups). Exclusion criteria were also applied in the instance that groups did not contain 6 active players. In some cases (N = 20 groups), groups were formed with less than six players, in order to avoid participants waiting too long for the game to start. After exclusions we collected data for N=57 full groups of 6 active players in each of the experimental conditions (N=171 groups in total).

Participant Level Exclusion Criteria

We excluded 310 participants from the individual level analyses who incorrectly answered the same comprehension question more than twice. We also implemented pre- and post-game attention checks in the form of a question within a survey that asked the participant to answer a certain way on a 5-point Likert scale. 38 participants failed the pre-game attention check and 64 participants failed the post-game attention check. These participants were excluded from the individual level analyses. After exclusions we collected data for n=818 participants who passed all comprehension and attention checks.

2. Pre-Registered Hypotheses

H1a: Adding a local option will reduce global cooperation

We expected to see less global cooperation in the Balanced condition than the Global Only condition. We believed that some groups would choose to cooperate locally instead of globally when given the option, considering that the local option required fewer participants to coordinate while giving an equivalent step return.

H1b: Adding a local option will increase total cooperation

We expected to see higher overall cooperation rates (less defection) in the Balanced condition than the Global Only condition. This hypothesis is somewhat counter to previous results, which have shown reduced contribution rates when participants have multiple options to invest in threshold public goods [2]. However, we considered that the underlying reason for the reduced contribution rates was a difficulty in coordination when facing multiple options with equivalent thresholds. In our game, the local option required fewer players to coordinate and therefore offered an alternate route to cooperation for groups where global cooperation has collapsed.

H2a: Increasing global payoffs will increase global cooperation
H2b: Increasing global payoffs will increase total cooperation

We expected higher global cooperation rates (a) and higher rates of overall cooperation (b) in the Global Boost condition than the Balanced condition due to the increased step returns [3].

H3a: Global cooperation will decline over the course of the game.
H3b: Total cooperation will decline (and defection will rise) over the course of the game.
H3c: We do not know whether local cooperation will decline over the course of the game.

Cooperation rates often decline in iterated threshold public goods games [4,5]. We expected to see similar declining trends in both global (a) and total (b) cooperation. We did not make a prediction either way about trends in local cooperation (c). On the one hand, we expected a decline in cooperation in general. However, we also expected that local cooperation rates might benefit from a decline in global cooperation, if participants chose to cooperate locally following a collapse of global cooperation.

H4a: Initial perceptions of social identity will be lower with the other group than the participant's own local group.
This was a sense check to verify that participants' perceptions of social identity were higher where they were more interdependent with others (as is the case in their own local vs other local group).

H4b: Initial perceptions of social identity will be different with the global and the participant's own local group.
Although some work has reported parochial biases, where smaller local groups are preferred over larger global groups [6–8], others have found the opposite [9]. This discrepancy may stem from individual differences in local vs global preferences [10], associated with political ideology [11,12]. Given the mixed findings, we predicted that we would find a difference in perceptions of social identity with local and global groups, but did not specify a direction for this difference.

H5: Initial perceptions of social identity will predict first round actions
We expected that participants who perceived greater social identity with the global (compared to the local) group would be initially more likely to contribute to the global (than the local) public good (c.f. Buchan et al., 2009, 2011; Chakravarty & Fonseca, 2017; Gallier et al., 2019; Wit & Kerr, 2002).

H6: Changes in perceptions of (local/global) social identity during the game will be associated with rates of successful (local/global) cooperation.
We expected perceptions of local/global social identity to be positively associated with rates of successful local/global cooperation (i.e. a 'performance-cohesion' effect, Beal et al., 2003).

H7: Perceptions of local and global social identity will decline over the course of the game.
Combining a decline in overall cooperation (and increase in defection) over the game (hypothesis 3b) and a performance-cohesion effect (hypothesis 6), we expected to see a decline in local and global social identities over the game.

3. Full Analysis Details

We used R (www.r-project.org, version 4.3.2) to carry out all analyses. Full statistical results for all analyses are provided in the Supplementary Information, and summarised in the Results section.

H1&H2. Differences in cooperation rates
To examine differences in cooperation rates across experimental conditions (addressing hypotheses 1 and 2), we calculated the aggregated rates of global, local and defection actions for each group over the entire game, yielding empirical distributions of group action rates for each condition. For each type of action (global, local, defect), we conducted pairwise comparisons between conditions using the Mann-Whitney U test [18] via the 'wilcox.test' function in the R 'stats' package [19]. This non-parametric test was chosen for its robustness in comparing

distributions without strong assumptions on the characteristics of those distributions, in contrast to the requirement of normality in the case of the t-test. Given the nature of our experimental data, which included ordinal measurements and potentially skewed distributions, the Mann-Whitney U test provided an appropriate analytical approach.

We followed a similar approach to compare threshold success rates between conditions, again employing Mann-Whitney U tests. We adapted the data aggregation approach described above to count threshold success rates at the global and local levels instead of action rates.

Data were aggregated at two levels to reflect the structure of the experimental design with a global group of 6 players and local groups of 3 players. In the case of global and defection actions, and for global thresholds, we compiled data at the 6-player global group; for local actions and thresholds, data were aggregated at the 3-player local group level.

We conducted a total of 11 Mann-Whitney U tests to assess differences across the various actions and thresholds. To mitigate the risk of Type 1 errors due to multiple comparisons, we applied a Holm-Bonferroni correction [20].

H3. Trends in Cooperation

To explore trends in cooperation rates over the 20 rounds of the experiment (addressing hypothesis 3), we counted the action rates for each group at each round. We used this data to fit three cumulative link mixed models (CLMMs) via the ‘clmm’ function in the ‘ordinal’ R package [21], specifying (1) global cooperation, (2) local (own) cooperation, and (3) defection as the respective dependent variables. Each model included ‘round’ as an independent variable as well as indicator variables for the Global Only and Global Boost conditions, with the Balanced condition serving as a reference. We included interaction terms for round*Global Only and round*Global Boost. We chose to use CLMMs as these models are well suited to detect trends where the dependent variable is ordinal, as is the case in this study.

The models analysing global cooperation and defection were aggregated at the 6-player global group level, incorporating a random intercept for each group to account for group level correlations. Similarly, the local model was aggregated at the 3-player local group level, including a random intercept for each local group.

In addition to assessing trends in cooperation, the CLMM analysis provided further support for comparisons between overall cooperation rates in hypotheses 1 and 2. Specifically, the coefficients of the indicator variables provided alternatives to the Mann-Whitney U tests to compare overall action rates in the Global Only vs Balanced and Global Boost vs Balanced conditions.

H4. Differences in Initial Perceptions in Social Identity.

We employed pairwise comparisons between the perceptions of social identity with the groups: global vs own local, global vs other local, and own local vs other local. To account for variation

across conditions we applied these tests separately with the data for each of the 3 experimental conditions, yielding a total of 9 comparisons.

We first tested our data for normality using a Shapiro-Wilk normality test. Finding that the data was highly non-normal in each case, we employed non-parametric Wilcoxon signed rank tests to make the pairwise comparisons. We also applied a Holm-Bonferroni correction to control the Type 1 family-wise error for multiple hypothesis tests.

In an exploratory analysis that was not pre-registered we investigated differences in initial social identity between experimental conditions. For each of the global, local (own) and local (other) groups, we compared initial perceptions pairwise between the Global Only vs Balanced, Global Only vs Global Boost and Global Boost vs Balanced conditions. We employed Mann-Whitney U tests to make these 9 comparisons, employing a Holm-Bonferroni correction for multiple hypothesis testing.

H5. Initial Perceptions of Social Identity and First Round Actions

To test whether initial social identity predicted first round actions, we used a logistic regression model via the ‘glm’ function in the R ‘stats’ package [19]. Specifically, we investigated whether differences in participants’ perceptions of social identity between the global group and their own local group predicted global cooperation over local cooperation. We coded a Boolean dependent variable with 1 for global and 0 for local cooperation, and considered an independent variable of the difference between the measured global and local social identity. We restricted data to conditions with a local option (Global Boost and Balanced), and only included participants who chose either global or local cooperation in round 1. We included an indicator variable for the Global Boost condition, with the Balanced condition serving as a reference.

H6. Performance-Cohesion Effect

To explore the relationship between social identity and group success we employed linear mixed effect regression models. At both the global and local group levels, we fit separate models with a dependent variable of the change in participants’ social identity in relation to that group between rounds 0 and 20 and an independent variable of the number of thresholds met by the participant’s group during the game. We included indicator variables for the Global Only and Global Boost conditions. We also included the initial social identity in relation to the group as a covariate to control for potential confounding relationships between initial perceptions and changes in perceptions. For example, the scale we employed has a maximum value and so positive changes are impossible if a participant initially rated a group at the maximum.

H7. Trends in Social Identity

To test for trends in social identity during the game we employed linear mixed effects regression models for each of the global, local (own) and local (other) groups, using the ‘mixed’ function in the ‘afex’ R package [22]. For each model, the dependent variable was the social identity in relation to that group, with an independent variable of the round number. We included indicator variables for the Global Only and Global Boost conditions, with the Balanced condition serving

as a reference category. In order to explore differences in trends across conditions, we also included interaction terms for Global Only*round and Global Boost*round.

4. Results: Detailed Statistical Analyses

Comparisons of Actions and Thresholds Met Across Conditions

We conducted 11 Mann-Whitney U analyses comparing (i) actions and (ii) thresholds met across conditions. To control for the family-wise type 1 error, we performed Holm-Bonferroni corrections.

Outcome	Comparison	Global Only	Balanced	Global Boost	W	p	p rank	adjusted p	Significance
Global Actions	Global Only vs Balanced	0.681	0.447		2491.5	9.03E-07	2	9.03E-06	***
Global Actions	Balanced vs Global Boost		0.447	0.607	1052	0.001184	5	0.008288	**
Global Actions	Global Only vs Global Boost	0.681		0.607	1971	0.04982	7	0.2491	NS
Local Actions	Balanced vs Global Boost		0.269	0.121	9139	1.02E-07	1	1.12E-06	***
Defection	Global Only vs Global Boost	0.319		0.271	1786.5	0.3599	9	1.0797	NS
Defection	Global Only vs Balanced	0.319	0.284		1766.5	0.4224	10	0.8448	NS
Defection	Balanced vs Global Boost		0.284	0.271	1645	0.9097	11	0.9097	NS
Global Threshold	Global Only vs Balanced	0.714	0.432		2405	9.44E-06	4	7.55E-05	***
Global Threshold	Balanced vs Global Boost		0.432	0.645	1067.5	0.001578	6	0.009468	**
Global Threshold	Global Only vs Global Boost	0.714		0.645	1839	0.2233	8	0.8932	NS
Local Threshold	Balanced vs Global Boost		0.258	0.0996	8657	1.46E-06	3	1.31E-05	***

*Table S1: Comparisons of actions and thresholds met across conditions. The table includes the Holm-Bonferroni corrected p-values. Corrected significance level denoted as *p < 0.05, **p < 0.01, ***p < 0.001. Note that the only test that changes significance level following the adjustment is the comparison of global action rates between the Global Only and Global Boost conditions.*

Trends of Actions During the Game

We fit 3 cumulative link mixed models (CLMMs) with predictor variables of i) number of global actions in a group; ii) number of local cooperative actions in a subgroup; iii) number of defect actions in a group. Explanatory variables for each were the round number, the experimental condition, and the interaction between round number and experimental condition.

	a. n(global)	b. n(local)	c. n(defect)
n(groups)	171	342	171
round	**-0.143*** **	**0.0316*** **	**0.123*** **
	(-0.163, -0.122)	(0.0155, 0.0477)	(0.104, 0.143)
	p<0.001	p<0.001	p<0.001
Global Only	**2.56*** **	-	0.173
	(1.58, 3.53)		(-0.641, 0.986)
	p<0.001		p=0.68
Global Boost	**1.39** **	**-1.32*** **	-0.299
	(0.43, 2.36)	(-2.04, -0.60)	(-1.110, 0.512)
	p=0.0048	p<0.001	p=0.47
round*Global Only	0.0183	-	0.0011
	(-0.0103, 0.0468)		(-0.0267, 0.0288)
	p=0.21		p=0.94
round*Global Boost	**0.0342* **	**-0.0564*** **	0.0038
	(0.0064, 0.0620)	(-0.0822, -0.0306)	(-0.0235, 0.0310)
	p=0.016	p<0.001	p=0.79
group intercept sd	2.48	-	2.00
subgroup intercept id	-	2.40	-

*Table S2: CLMMs exploring a) global cooperation b) local cooperation c) defection over rounds in each condition. Each column shows a separate model. Note that local cooperation was not an action available to participants in the Global Only condition. Global cooperation (a) and defection (c) models were aggregated at the 6 player global group level, with random intercepts for global groups. The local models (b) were aggregated at the 3 player local group level, with random intercepts for local groups. For each independent variable, coefficient estimates are reported with confidence intervals in parentheses and p-values. Significance levels are denoted as follows: *p < 0.05, **p < 0.01, ***p < 0.001.*

In order to test for a trend in local cooperation in the Global Boost condition we summed the coefficients for main effect of round and the round*Global Boost interaction in Table S2. This gives a trend coefficient of 0.0316 - 0.0564 = -0.0248. The standard error of the combined

coefficient was calculated, accounting for the variance-covariance structure of the parameters, as

$$se = \sqrt{var(round) + var(round * Global\ Boost) - 2cov(round, round * Global\ Boost)}.$$

Given this standard error, significance was calculated using a Wald test, finding p=0.02.

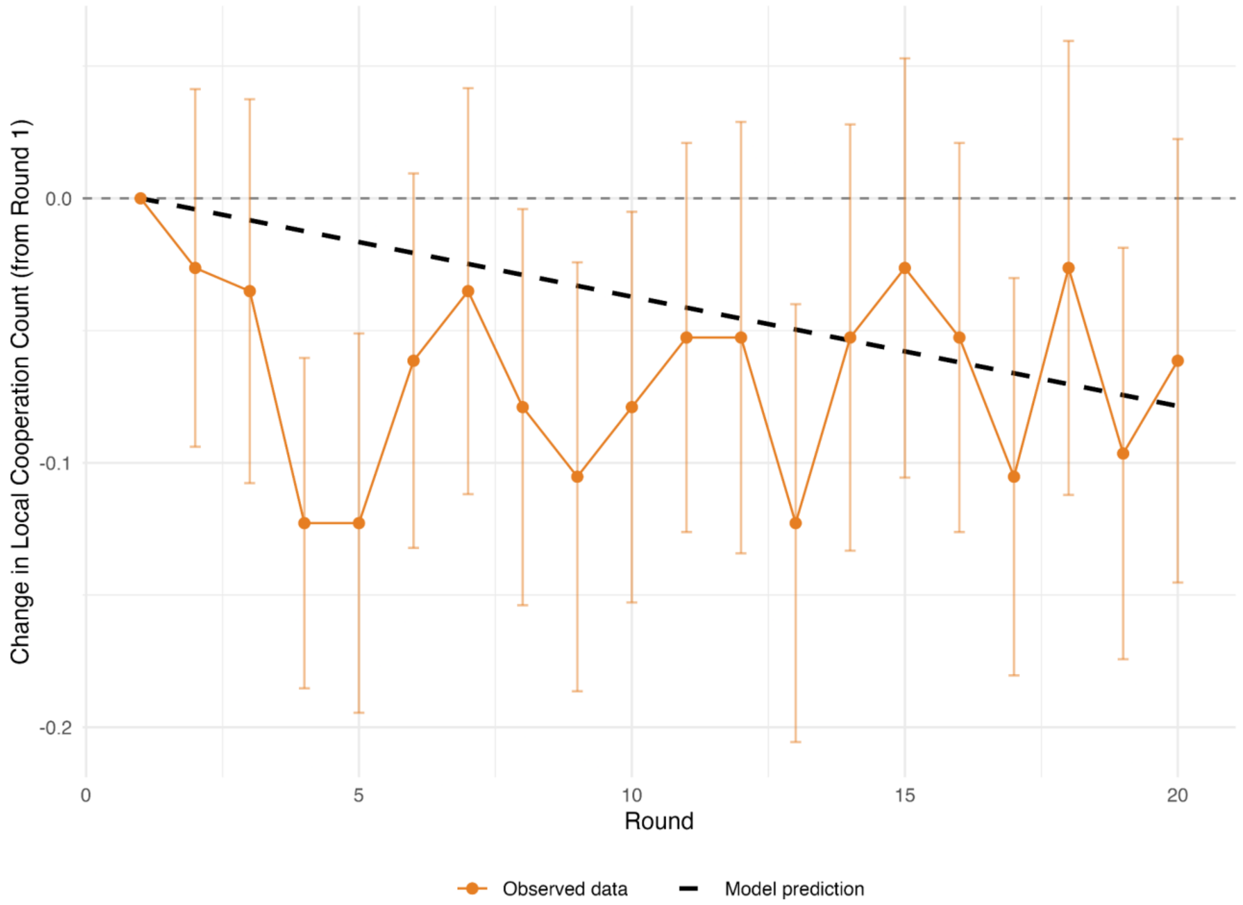

Figure S1: Relative trend in local cooperation actions in the Global Boost condition. Orange points show the mean number of local cooperation actions within groups, relative to the number of local cooperation actions by those groups in round 1. Error bars show the standard error. The black dotted line shows the CLMM model prediction.

Comparison of Initial Perceptions of Social identity.

In order to assess differences in how participants perceived their identity with the global, local (own) and local (other) groups, we compared these perceptions within each experimental condition using paired Wilcoxon signed rank tests. We found significant differences and a consistent ordering of initial perceptions of social identity with the local group highest, followed

shortly by the global group and with the other local group rated much lower. We employed a Holm-Bonferroni correction to adjust significance levels to account for multiple hypothesis tests.

Condition	Comparison	n(participants)	Initial identity mean (sd) [se]			W	p-value	p-rank	adjusted p-value
			**Local**	**Global**	**Other**				
Global Only	Global vs Other	228		90.5 (13.1) [0.868]	62.6 (23.4) [1.55]	24400	2.14E-37	5	1.07E-36
Global Only	Local vs Other	228	91.9 (12.6) [0.834]		62.6 (23.4) [1.55]	24300	1.53E-33	6	6.12E-33
Global Only	Global vs Local	228	91.9 (12.6) [0.834]	90.5 (13.1) [0.868]		4920	9.81E-03	9	9.81E-03
Balanced	Global vs Other	304		89.9 (11.5) [0.660]	47.2 (27.3) [1.57]	45600	1.26E-50	1	1.13E-49
Balanced	Local vs Other	304	93.4 (11.6) [0.665]		47.2 (27.3) [1.57]	45300	3.36E-48	2	2.69E-47
Balanced	Global vs Local	304	93.4 (11.6) [0.665]	89.9 (11.5) [0.660]		6050	1.82E-09	7	5.46E-09
Global Boost	Global vs Other	286		90.7 (11.9) [0.704]	50.1 (27.0) [1.60]	40300	2.94E-46	3	2.06E-45
Global Boost	Local vs Other	286	93.2 (10.9) [0.644]		50.1 (27.0) [1.60]	40100	2.04E-45	4	1.22E-44
Global Boost	Global vs Local	286	93.2 (10.9) [0.644]	90.7 (11.9) [0.704]		6870	6.95E-05	8	1.39E-04

Table S3: Comparisons of initial perceptions of identity within each condition between the global group, participant's own local group and the other local group. Comparisons were made using paired Wilcoxon signed ranked tests. A Holm-Bonferroni correction was applied, with the adjusted p-values shown.

We also explored differences in initial perceptions in relation to each group (global/local/other) across experimental conditions using Wilcoxon rank-sum tests. We found that most pairwise comparisons were not significant, and only found a significant difference in the comparison of perceptions of the other local group in i) the Global Only vs Balanced condition and ii) the Global Only vs Global Boost conditions.

Identity	Comparison	U	p	p rank	adjusted p-value
Global	Global Only vs Balanced	32700	0.236	4	1

Global	Global Only vs Global Boost	31600	0.542	7	1
Global	Global Boost vs Balanced	42300	0.558	8	1
Local	Global Only vs Balanced	36300	0.311	5	1
Local	Global Only vs Global Boost	34000	0.393	6	1
Local	Global Boost vs Balanced	43800	0.854	9	0.854
Other	Global Only vs Balanced	22400	2.77E-12	1	2.49E-11
Other	Global Only vs Global Boost	23000	7.91E-09	2	6.33E-08
Other	Global Boost vs Balanced	40500	0.156	3	1

Table S4: Comparisons of initial perceptions of identity between experimental conditions for the global group, the participant's own local group and the other local group. Pairwise comparisons were made using Wilcoxon rank-sum test, with a Holm-Bonferroni correction for multiple hypothesis tests. The only significant differences were in perceptions of the other local group between i) Global Only and Balanced and ii) Global Only and Global Boost conditions.

Trends in Perceptions of Social Identity

We fit mixed effects linear models to explore trends in social identity with a) the global group, b) participants' own local group, and c) the other local group.

	a. global identity	b. local identity	c. other identity
n(participants)	818	818	818
Fixed Effects			
Intercept	**89.6*** **	**90.0*** **	**44.9*** **
	(86.8, 92.3)	(87.5, 92.6)	(42.0, 47.8)
	p<0.001	p<0.001	p<0.001
round	**-0.759*** **	**-0.897*** **	**-0.740*** **
	(-0.857, -0.660)	(-1.007, -0.786)	(-0.879, -0.601)
	p<0.001	p<0.001	p<0.001
Global Only	0.256	0.982	**18.5*** **
	(-3.868, 4.380)	(-2.911, 4.875)	(14.1, 22.9)
	p=0.903	p=0.622	p<0.001
Global Boost	0.601	-0.613	3.90
	(-3.378, 4.580)	(-4.324, 3.098)	(-0.27, 8.06)
	p=0.767	p=0.746	p-0.0675

round*Global Only	**0.399*** **	**0.404*** **	**0.725*** **
	(0.248, 0.550)	(0.236, 0.572)	(0.512, 937)
	p<0.001	p<0.001	p<0.001
round*Global Boost	**0.227** **	-0.0482	0.0358
	(0.086, 0.369)	(-0.2064, 0.1100)	(-0.1638, 0.2353)
	p=0.0017	p=0.551	p=0.726
Random Effects			
participant intercept sd	14.1	15.1	24.1
participant*round sd	-	-	1.09
subgroup intercept sd	-	4.21	3.57
subgroup*round sd	-	-	-
group intercept sd	8.11	4.98	-
group*round sd	-	-	-

*Table S5: Mixed effects linear regression models to explore trends in social identity over the game. Trends were analysed in relation to the a) global, b) local, and c) other groups. Data was included from all experimental conditions with indicator variables for the Global Only and Global Boost conditions, with the Balanced condition as reference. Fixed effect coefficient estimates are shown with confidence intervals in parentheses and p-values. Random effect standard deviations are shown. We began with maximal multilevel models and removed random effects until the fit converged and was non-singular. Significance levels are denoted as follows: *p < 0.05, **p < 0.01, ***p < 0.001.*

Performance Cohesion Effect

To explore associations between changes in social identity and group success we fit linear mixed effects models at both the global and local group level. For each model, we included a control variable in the initial perceptions of social identity, finding a negative association such that those participants with high initial ratings of social identity tended to show a larger decrease in those perceptions during the game (global model initial identity coefficient = -0.463, p<0.001; local model initial identity coefficient = -0.457, p<0.001).

	**a. change in global identity**	**b. change in local identity**
n(participants)	818	590
intercept	8.50	16.3
	(-2.92, 19.95)	(-1.9, 34.7)

	p=0.146	p=0.082
number of global thresholds	**2.09*** **	-
	(1.84, 2.35)	
	p<0.001	
number of local thresholds	-	**1.35*** **
		(0.91, 1.80)
		p<0.001
initial global identity	**-0.463*** **	-
	(-0.584, -0.342)	
	p<0.001	
initial local identity	-	**-0.457*** **
		(-0.648, -0.267)
		p<0.001
Global Only	-2.54	-
	(-6.78, 1.71)	
	p=0.245	
Global Boost	-2.89	2.13
	(-6.84, 1.05)	(-2.89, 7.18)
	p=0.155	p=0.409
Global group random intercept sd	5.35	9.71
Local group random intercept sd	**-**	4.48

*Table S6: Performance-cohesion effect. At both the a) global and b) local group level, changes in group identity were positively associated with the number of rounds the respective group met the threshold. We began with a maximal multilevel model and removed random effects in a predetermined order until the model successfully converged. Significance levels are denoted as follows: *p < 0.05, **p < 0.01, ***p < 0.001.*

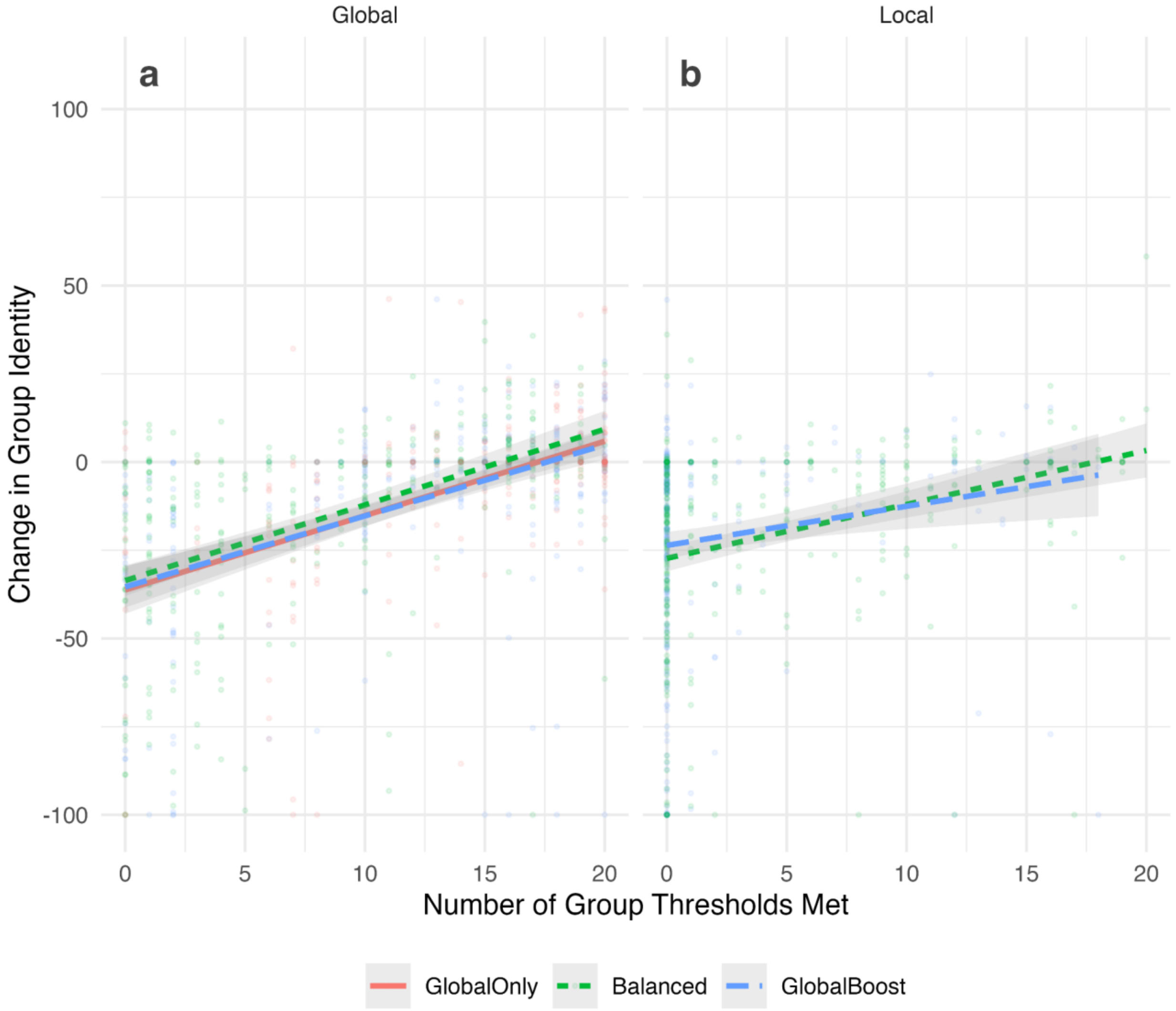

Figure S2: Performance cohesion effect at the global and local level in all conditions. a) The number of global thresholds met during the game is positively associated with changes in perception of global group identity over the game. b) The number of local thresholds met is positively associated with increases in perception of local group identity. Scatter points show results from each individual participant. Lines show simple linear regression models with shaded 95% confidence intervals.

Predicting First Round Actions From Social Identity

We fit a logistic regression to predict first round actions with an independent variable of the difference between reported initial perceptions of global and local social identity. We only considered data where the first round action was either global or local cooperation, in order to give a relevant binary response variable. We included data from the Balanced and Global Boost condition, where local cooperation was an option. We also included an indicator variable for the Global Boost condition to control for the influence of different incentive structures.

	p(global|global or local)
n(participants)	490
Intercept	**1.25*** **
	(0.94, 1.58)
	p<0.001
global minus local identity	**0.0253***
	(0.0020, 0.0504)
	p=0.0408
Global Boost	**0.933** **
	(0.386, 1.512)
	p=0.0011
Global Boost*global minus local identity	0.0367
	(-0.0052, 0.0802)
	p=0.0917

*Table S7: Predicting first round actions from initial social identity. Table shows results of a logistic regression with data from the Balanced and Global Boost conditions. Data was restricted to participants who chose either global or local cooperation in the first round, such that the dependent variable in the model is p(global|global or local). Significance levels are denoted as follows: *p < 0.05, **p < 0.01, ***p < 0.001.*

5. Further Pre-registered Analyses

In the original pre-registration we made a series of hypotheses in relation to the Balanced condition only. We subsequently posted a second pre-registration to expand the game to the Global Only and Global Boost conditions. We report the results of the hypotheses for the first registration here, where these were not included in the main paper.

Mutual Dependence and Conflict

In the course of these analyses we use some variables not described in the main text. These variables are described below in the same way as in the pre-registration.

To measure perceptions of mutual dependence and conflict, respectively, we used selected items from the Situational Interdependence Scale [23]. Responses to questions were obtained using a 5 point Likert scale, where 1 = “Completely Disagree” and 5 = “Completely Agree”. For mutual dependence, we used the following questions: *(1) MD Q1: ”What each of us does in this*

situation affects the other.; (2) MD Q2: "Whatever each of us does in this situation, our actions will not affect the other's outcomes." For perceptions of conflict we used the following questions: *(1) Conflict Q1: "Our preferred outcomes in this situation are conflicting."; (2) Conflict Q2: "We can both obtain our preferred outcomes."*

Combined measures of mutual dependence and conflict, respectively, were obtained by combining the numerical values of the responses to the questions as follows (note that MD Q2 is negatively coded): Mutual Dependence = MD Q1 + (5 - MD Q2). This yields 3 DVs for local (own), local (other) and global perceived mutual dependence, which are positive integers that can take values between 1 and 9. We followed the same procedure to combine the responses to generate a measure of perceived conflict, and note that Conflict Q2 is negatively coded, such that Conflict = Conflict Q1 + (5 - Conflict Q2). As with mutual dependence, this yielded 3 DVs for local (own), local (other) and global perceived conflict, which are again positive integers that can take values between 1 and 9.

Hypotheses and Results

Hypothesis 1: Initial perceptions of mutual dependence, conflict and social identity will be different with members of a local compared to members of a global group. Based on our pilot data, we are agnostic as to the direction of this effect.

Hypothesis 1a: Initial perceptions of mutual dependence and social identity will be higher for the participant's own local group than for the other local group, and perceptions of conflict will be higher for the other local group than for the own local group. *This is just a sense check.*

Using data from the Balanced condition only, we compared the initial perceptions in social identity, mutual dependence and conflict between a) the global and local group and b) the participant's local and the other local group. The data was highly skewed and non-normal, and so we used non-parametric paired Wilcoxon signed rank tests. We also applied a Holm-Bonferroni adjustment to correct for multiple hypothesis tests.

Perceptions of identity and mutual dependence were lower for the other local group than the own local group (as predicted), and perceptions of conflict with the other local group were higher, compared to one's own local group (as predicted). Perceptions of identity were higher for participants' own local group than the global group, though people perceived more mutual dependence with the global group than their local group. Although perceptions of conflict were higher for the global than for the local group, this difference was not statistically significant.

Condition	Comparison	n(participants)	Initial Mean (SD)			W	p-value	p-rank	adjusted p-value
			Global	Local	Other				
Balanced	Global vs Local Identity	304	89.9 (11.5)	93.4 (11.6)		6050	1.82E-09	4	5.46E-09

Balanced	Global vs Local MD	304	9.11 (1.02)	7.91 (1.21)		4980	1.00E-03	5	0.002
Balanced	Global vs Local Conflict	304	3.31 (1.68)	3.22 (1.82)		8840	2.50E-01	6	0.25
Balanced	Local vs Other Identity	304		93.4 (11.6)	47.2 (27.3)	45300	3.36E-48	1	2.02E-47
Balanced	Local vs Other MD	304		7.91 (1.21)	7.12 (1.64)	9580	7.43E-17	3	2.97E-16
Balanced	Local vs Other Conflict	304		3.22 (1.82)	4.43 (1.82)	1470	7.15E-20	2	3.58E-19

Table S8: Comparisons of initial perceptions of group identity, mutual dependence and conflict between a) global and local group b) participant's local and other local group. Considering data from the Balanced condition only. Significant effects were found in all pairwise comparisons except for initial perceived conflict between the local and global group.

Hypothesis 2: Initial perceptions of mutual dependence at local and global scales will be positively correlated with perceptions of social identity at the same scales.

Hypothesis 3: Initial perceptions of conflict at local and global scales will be inversely associated with perceptions of social identity at the same scales.

We hypothesised that there would be correlations in perceptions of social identity and mutual dependence, and social identity and conflict. Specifically, we analysed correlations in perceptions in relation to a) the global group and b) the participants' own local group. We performed Spearman's rank correlations of mutual dependence and social identity, and conflict and social identity. We found no significant correlations at either the local or the global group scale. We note that the variance of perceptions in relation to each group were small, i.e. there was little variation within which to measure correlations, so this analysis may have been underpowered. This analysis was restricted to data from the Balanced condition.

Condition	Hypothesis	n(participants)	Correlation, rho (Confidence Interval)	S	p-value	p-rank	adjusted p-value
Balanced	Initial Local MD ~ Local Identity	304	0.0591 (-0.0537, 0.1705)	4.41E+06	0.304	3	0.608
Balanced	Initial Global MD ~ Global Identity	304	0.0857 (-0.0271, 0.1963)	4.28E+06	0.136	1	0.544
Balanced	Initial Local Conflict ~ Local Identity	304	-0.00619 (-0.11860, 0.10638)	4.71E+06	0.914	4	0.914
Balanced	Initial Global Conflict ~ Global Identity	304	-0.0787 (-0.1895, 0.0341)	5.05E+06	0.171	2	0.513

Table S9: Spearman's rank correlations between a) group identity ~ mutual dependence and b) group identity ~ conflict for i) initial perceptions in relation to the local group, ii) initial perceptions in relation to the global group.

Hypothesis 4: Changes in perceptions of (local/global) interdependence, conflict, and social identity between the start and the end of the game will be associated with rates of successful (local/global) cooperation during the game.

We explored a performance-cohesion effect with measures of perceptions of social identity, mutual dependence and conflict. We conducted 6 GLMMs, to respectively predict 6 dependent variables of change in perception of identity, mutual dependence and conflict at the global and local levels. The change in perception for each participant was defined as the value of the perception measured after the game minus the value of the same perception measured before the game began. We included independent variables of the number of thresholds met at the relevant group level. We also included the initial value of the relevant perception as a covariate as an attempt to control for any effect that initial perceptions may have on the subsequent group success, as well other effects such as regression to the mean. These analyses were restricted to data from the Balanced condition.

We included random effects in relation to the participants group and subgroup (their own local group). We began with maximal multilevel models and removed random effects until the model successfully converged, as described in the pre-registration.

We found performance-cohesion effects whereby success was correlated with positive changes in social identity at the global and local levels, positive changes in mutual dependence at the global level, and negative changes in conflict at the global and local levels. We did not find a significant relationship between group success and changes in mutual dependence at the local level.

	a. Change in Social Identity		b. Change in Mutual Dependence		c. Change in Conflict	
	i) Global	ii) Local	i) Global	ii) Local	i) Global	ii) Local
n(participants)	304	304	304	304	304	304
Fixed Effects						
Intercept	6.83	**30.3****	**2.54*****	**4.21*****	**2.45*****	**2.04*****
	(-13.61, 24.47)	(8.1, 52.7)	(1.43, 3.67)	(3.29, 5.12)	(1.94, 2.96)	(1.60, 2.47)
	p=0.515	p=0.00796	p<0.001	p<0.001	p<0.001	p<0.001
Initial Perception	**-0.449*****	**-0.611*****	**-0.389*****	**-0.545*****	**-0.498*****	**-0.451*****
	(-0.672, -0.228)	(-0.846, -0.378)	(-0.525, -0.255)	(-0.662, -0.434)	(-0.617, -0.379)	(-0.561, -0.342)
	p<0.001	p<0.001	p<0.001	p<0.001	p<0.001	p<0.001
Number of thresholds met	**2.14*****	**1.45*****	**0.0419****	0.0159	**-0.0484****	**-0.0360***

	(1.74, 2.55)	(0.94, 1.96)	(0.0173, 0.0665)	(-0.0074, 0.0394)	(-0.0786, -0.0182)	(-0.0700, -0.0020)
	p<0.001	p<0.001	p=0.00135	p=0.185	p=0.00177	p=0.0486
Random Effects						
Intercept*group_id sd	3.97	-	0.420	-	-	0.247
Initial Perception*group_id sd	-	-	-	-	-	0.0343
Number of thresholds met*group_id sd	-	-	-	-	-	-
Intercept*subgroup_id sd	-	11.49	-	0.305	-	0.000477
Initial Perception*subgroup_id sd	-	-	-	-	-	0.0780
Number of thresholds met*subgroup_id sd	-	-	-	-	-	0.0200

*Table S10: Performance-cohesion effect. Each column represents a single mixed effects model, with dependent variables of changes in perceptions of a) social identity b) mutual dependence c) conflict with the i) global and ii) local group. Fixed effect coefficient estimates are shown with confidence intervals in parentheses and p-values. Random effect standard deviations are shown. Significance levels are denoted as follows: *p < 0.05, **p < 0.01, ***p < 0.001.*

Hypothesis 5: Perceptions of local and global social identity will decline over the course of the game.

We predicted that global and local social identity would decline over the course of the game. To test this hypothesis, we fit 2 mixed effects linear models to predict changes in 1) global and b) local identities. We found significant negative coefficients on the round number in both cases. We began with a maximal multilevel model and removed random effects until the models successfully converged, as described in the pre-registration. Data was restricted to the Balanced condition.

	a. Global identity	b. Local identity
n(participants)	304	304
Fixed Effects		

Intercept	**89.6*** **	**90.1*** **
	(86.8, 92.4)	(88.5, 91.7)
	p<0.001	p<0.001
round	**-0.759*** **	**-0.892*** **
	(-0.857, -0.660)	(-1.079, -0.705
	p<0.001	p<0.001
Random Effects		
participant intercept sd	14.6	8.99
participant*round sd	-	0.931
subgroup intercept sd	-	3.46
subgroup*round sd	-	0.743
group intercept sd	13.9	-
group*round sd	-	-

*Table S11: Declining trends in a) global and b) local identity over the rounds of the game. Each column represents a mixed effects linear model. Fixed effects coefficient estimates are reported along with confidence intervals in parentheses and p-values. Random effect standard deviations are shown. We began with maximal multilevel models and removed random effects until the fit converged and was non-singular. Significance levels are denoted as follows: ***p < 0.001.*

Hypothesis 6: People who perceive more mutual dependence/less conflict/greater social identity, with the local (compared to the global) group will be initially more likely to contribute to the local (than the global) public good.

We fit a logistic regression model to predict whether a participant's first action was global or not, with data restricted to participants whose first action was either global or local, and in the Balanced condition only (n=253 participants). For prediction variables, we included the difference in initial perceptions between the global and local group of i) social identity ii) mutual dependence and iii) conflict. We did not find significant effects for any of the initial perception measures with 2-sided tests.

If we consider one-sided tests, then the social identity coefficient was significant with p<0.05. In the main article we perform a higher powered analysis including data from both Balanced and Global Boost conditions, and we do find a significant positive association with differences in social identity and first round actions.

We checked for colinearity in independent variables by computing the variance inflation function, finding values less than 1.02 for all measures, indicating a low degree of collinearity.

	p(global)
n(participants)	253
intercept	**1.25*** **
	(0.94, 1.59)
	p<0.001
initial (global - local) identity	0.0243
	(0.0009, 0.0498)
	p=0.0516
initial (global - local) mutual dependence	-0.00646
	(-0.276, 0.270)
	p=0.963
initial (global - local) conflict	0.0839
	(-0.06915, 0.23810)
	p=0.282

*Table S12: Logistic regression model to predict whether each participant's action in the first round was global, given that their action was either global or local. Coefficient estimates are reported along with confidence intervals in parentheses and p-values. The significant intercept indicates that global investment actions are overall more likely than local investment actions in round 1. Significance levels are denoted as follows: ***p < 0.001.*

Hypothesis 6a: People who perceive less mutual dependence/more conflict/less social identity will be initially more likely to defect than to contribute to the local or global public good.

We fit a logistic regression model to predict whether the first action was to defect, given a participant's ratings of the local and global social identity, mutual dependence and conflict. We restricted the data to the Balanced condition and to participants whose first action was either defect or to cooperate globally or locally, meaning that we removed 7 participants who did not choose any option within the time limit of 30 seconds in the first round.

We did not find evidence that perceptions of identity, mutual dependence or conflict in relation to the global or local groups are predictive of defection in the first round. We checked for collinearity in independent variables by computing the variance inflation function, finding values less than 2 for all measures, indicating a low degree of collinearity.

As explained in the pre-registration, we suspected that this analysis might be underpowered and decided to proceed while noting that this is not a main hypothesis.

	p(defect)
n(participants)	297
intercept	-1.18
	(-5.71, 3.04)
	p=0.594
local identity	0.000370
	(-0.027410, 0.035119)
	p=0.981
local mutual dependence	-0.215
	(-0.506, 0.089)
	p=0.154
local conflict	-0.163
	(-0.392, 0.049)
	p=0.144
global identity	0.0159
	(-0.0147, 0.0485)
	p=0.320
global mutual dependence	-0.0299
	(-0.3899, 0.3527)
	p=0.874
global conflict	0.115
	(-0.104, 0.333)
	p=0.298

Table S13: A logistic regression model to predict the probability (the log odds) of defecting in the first round, with no significant effects found in perceptions of local/global identity, mutual

dependence or conflict. Coefficient estimates are reported along with confidence intervals in parentheses and p-values.

Hypothesis 7: The outcome of the game in each round will predict participants' next actions.

We investigated whether the group outcome of a game in a round was predictive of participant's actions in the next round. The outcome of a game, from a focal participant's perspective, was defined as the number of global investors, number of local investors in the participant's own local group, and the number of defectors in the global group. We included squared transformations of these variables, to account for non-linear responses e.g. a player may be likely to invest globally if the group almost met the threshold, but perhaps less likely if the group overshot the threshold. We also included a variable for the participant's last action, as we expected a degree of inertia in actions. And we include a variable for the round number, to account for changing preferences over the game.

We removed data where participants chose to invest in the "other" local group (0.76% of actions) or did not make a choice within the time limit on the decision page (0.22% of actions). Following this, the dependent variable of the participant's next action was categorical with options "global", "local" or "defect". In order to model this multi-class categorisation problem we used logistic regression with nested dichotomies. We first fit a logistic regression model to predict whether a participant would choose "global" or not. And then, considering only the data where a participant did not choose global, we fit a logistic regression model to predict local cooperation vs defection. We began with maximal models that included random effects, and reduced the random effects until the models converged. In both the global and local cases the model did not converge until we reduced the model to include only fixed effects.

As predicted, participants respond to the outcome of the game in the last round, with significant coefficients related to the number of players in the group who cooperated globally and locally. The round coefficient is negative for both models, but only significant in the case of the local action model. We also see an inertia effect, whereby participants are more likely to cooperate at the global and local level if they cooperated at that same level in the last round.

	a. p(global)	b. p(local|not global)
n(participants)	304	304
n(actions)	5738	3200
Fixed Effects		
intercept	**-3.48*** **	**1.21*** **
	(-3.97, -3.00)	(-1.74, -0.70)
	p<0.001	p<0.001
round	-0.00915	**-0.0423*** **

	(-0.02183, 0.00353)	(-0.0608, -0.0239)
	p=0.157	p<0.001
last action was global	**1.89*** **	**1.04*** **
	(1.73, 2.04)	(0.80, 1.29)
	p<0.001	p<0.001
last action was local	0.0208	**2.63*** **
	(-0.1878, 0.2282)	(2.42, 2.84)
	p=0.845	p<0.001
n(global)	**1.83*** **	-0.00721
	(1.61, 2.05)	(-0.21796, 0.20464)
	p<0.001	p=0.947
n(local)	**-0.332* **	**2.29*** **
	(-0.663, 0.002)	(1.91, 2.68)
	p=0.050	p<0.001
n(defect)	0.114	-0.172
	(-0.086, 0.315)	(-0.423, 0.079)
	p=0.263	p=0.179
n(global) squared	**-0.274*** **	-0.0453
	(-0.314, -0.235)	(-0.0919, 0.0007)
	p<0.001	p=0.0549
n(local) squared	0.101	**-0.891*** **
	(-0.078, 0.273)	(-1.056, -0.728)
	p=0.260	p<0.001
n(defect) squared	0.00444	0.00554
	(-0.04529, 0.05341)	(-0.05209, 0.06215)
	p=0.860	p=0.849

Table S14: Logistic regression models with nested dichotomies to predict a) The probability (log odds) that a participant will invest in the global group, and b) Given that the investment is not global, the probability (log odds) that the participant invests in the local group instead of

*defecting. Participants respond to the group outcome in the last round, with significant coefficients in the number of players who took each action. We also see an inertia effect in that participants have increased likelihood to take the same cooperative action as in the last round. There is also declining global and local cooperation over time, with a negative round coefficient. Fixed effect coefficient estimates are shown with confidence intervals in parentheses and p-values. Random effect standard deviations are shown. We began with maximal multilevel models and removed random effects until the fit converged and was non-singular. Significance levels are denoted as follows: *p < 0.05, ***p < 0.001.*

6. Further Exploratory Analyses

Action Distributions Timeseries

In the main text we show timeseries for the actions taken by groups over rounds, aggregated across each experimental condition.

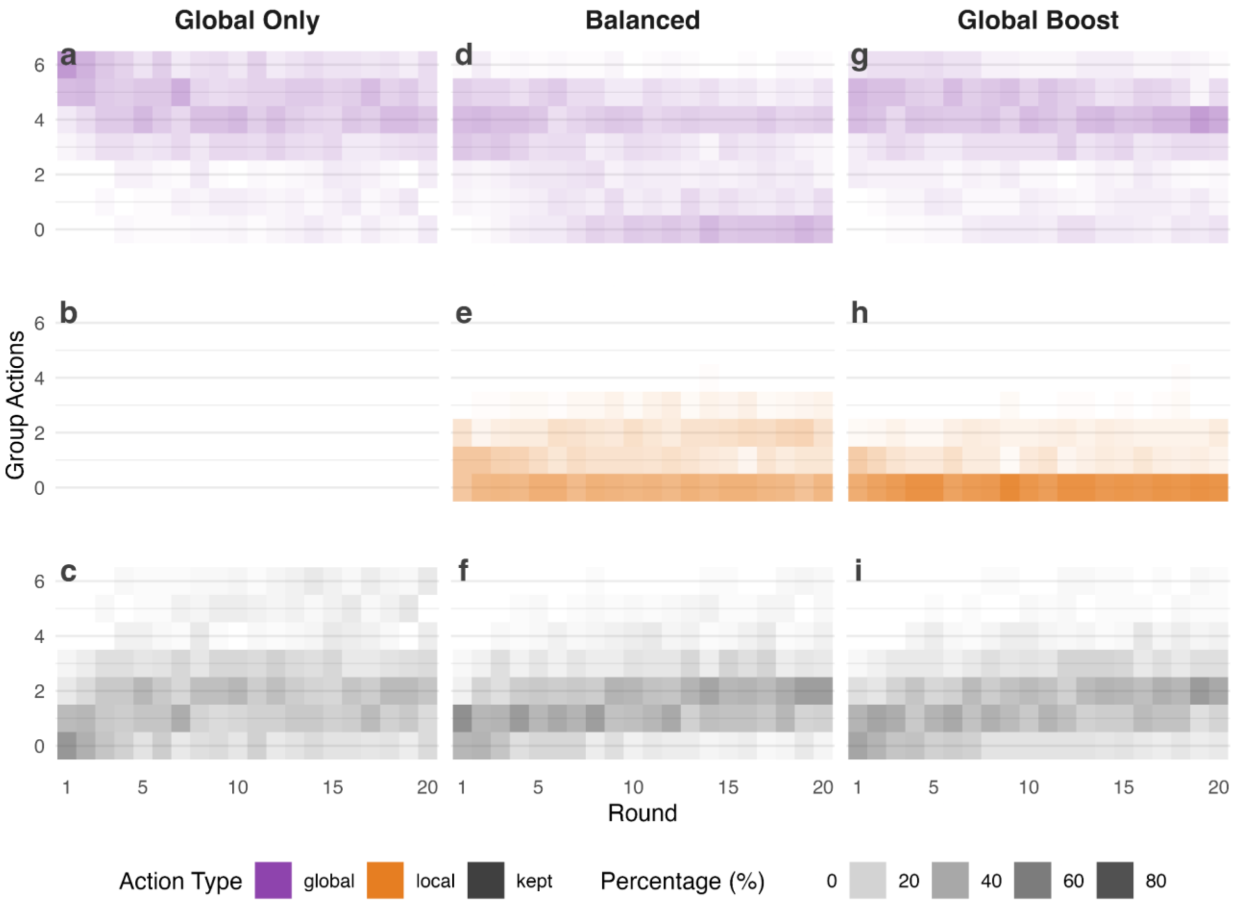

Figure S3: Action distribution timeseries across conditions (left to right) and action type (top to bottom). For each round (x-axis), the relative proportion of groups with each action count is shown as a shaded box.

Group Dynamics

In the main paper we show trends of global, local, and defection actions within groups over rounds. Figure S3 more thoroughly displays the action distributions within groups, with the number of global and local cooperation actions. At the early stages of the game (rounds 1-5), distributions are unimodal. At the later stages of the game (round 16-20), distributions become more multimodal with modal peaks around Nash Equilibria. In the Balanced condition, there are clear peaks at the global cooperative equilibrium (4 global cooperators and 0 local cooperators) and the subgroup local cooperative equilibrium (0 global cooperators and 2 local cooperators). In the Global Boost condition, the global cooperative equilibrium is more pronounced, reflecting the greater incentives. We further explored the group dynamics through a one-step state space dynamical analysis in Figure S4, with arrows showing the mean direction of movement within the space from one round to the next, and a contour plot showing the distribution of actions within groups over the entire experiment. There are attraction basins towards the Nash Equilibria, indicating path dependency in group behaviours.

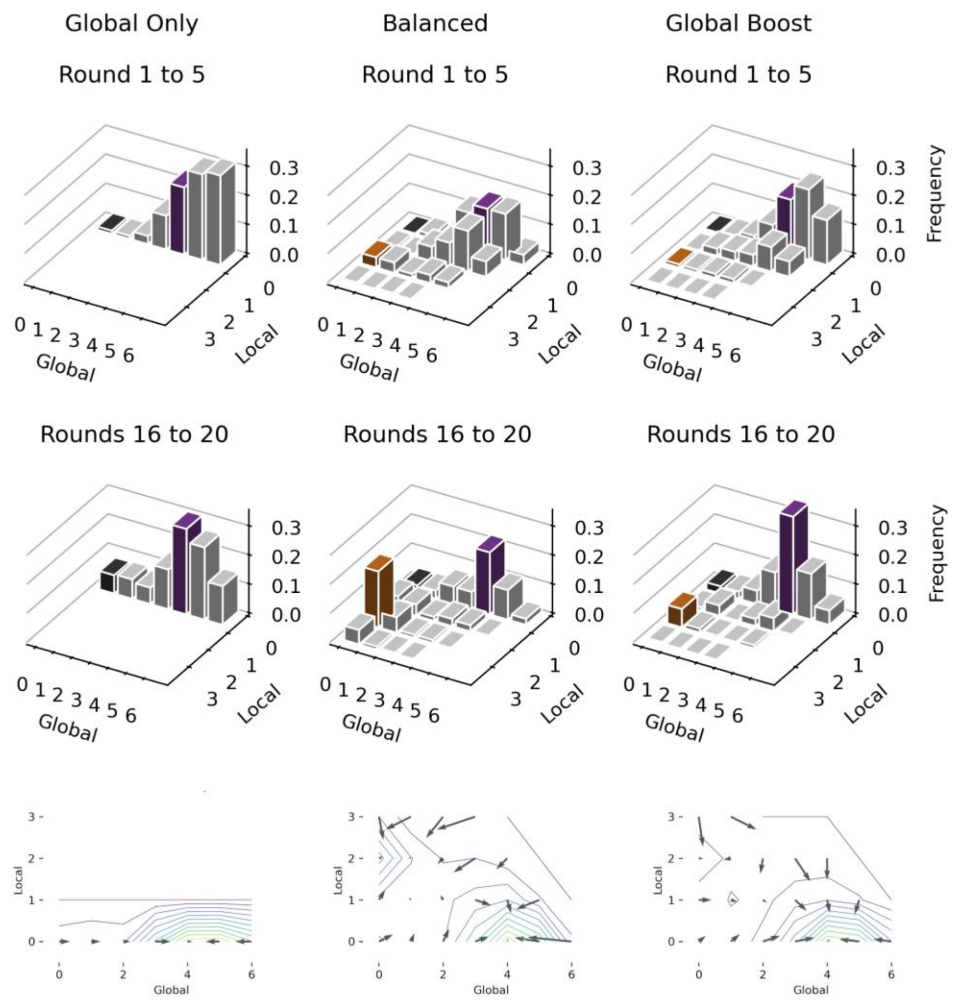

Figure S4: Group dynamics of round outcomes in groups. (top two rows) Distributions of the number of global cooperators and number of local cooperators in subgroups from the perspective of focal individuals. The global (purple), local (orange) and defection (black) Nash Equilibria are highlighted. (top row) Distributions of round outcomes in groups in rounds 1 to 5 appear unimodal, and not strongly centered around Nash Equilibria. (middle row) Distributions of round outcomes in rounds 16 to 20 are bimodal with modes centered on Nash Equilibria. (bottom row) One-step round dynamics indicate attraction basins around Nash Equilibria.

Intergroup Correlation Between Social Identity and Conflict Mutual Dependence

We investigated correlations between perceptions of social identity and measures of perceptions of mutual dependence and conflict, including data in relation to all types of groups and from all conditions, and measurements at the start and end of the game. We found a

positive correlation between social identity and mutual dependence, and a negative correlation between social identity and conflict.

	Analysis	n(observations)	Correlation, rho (Confidence Interval)	S	p-value
Balanced	Identity ~ Mutual Dependence	1026	0.183 (0.159,0.207)	3.18E+10	<2.2E-16
Balanced	Identity ~ Conflict	1026	-0.269 (-0.325, -0.211)	4.93E+10	<2.2E-16

Table S15: Correlations between perceptions of social identity and mutual dependence, and perceptions of social identity and conflict. This includes data in relation to all group types (local, global, other), across all experimental conditions and questions surveyed at the beginning and end of the game.

Behavioural Convergence

As an exploratory analysis we investigated whether behaviour converged to a stable state over the game. We fit CLMMs to action trends in each condition, with the addition of interaction terms between round and a categorical variable for the early phase of the game (rounds 1-10), with the later phase of the game (rounds 11-20) serving as a reference category. A non-significant round coefficient indicates that there was not a significant trend in the later phase (the last 10 rounds reference category). We fit a total of 8 models across conditions (Global only, Balanced, Global Boost) and action types (global, local, defect); with no model for local actions in the Global Only condition.

In most cases we did not detect a significant trend in the later phase of the game, with a non-significant round coefficient (Tables S16 - S18). The exceptions were global actions in the Balanced condition and defect actions in the Global Boost condition, which found significant trends in the second half of the game. In all models, the round coefficients in the later phase of the game were much smaller than the round:early phase coefficient. This pattern is consistent with greater behavioural stability in the later phase of the game than the earlier phase.

	a. Global Only	b. Balanced	c. Global Boost
	n(global)	n(global)	n(global)
n(groups)	57	57	57
Fixed Effects			

round	-0.0346	**-0.0639***	-0.0479
	(-0.0898, 0.0205)	(-0.119, -0.00927)	(-0.102, 0.00614)
	p = 0.218	p = 0.0219	p = 0.0823
early phase	**2.53*****	**1.85*****	**1.47****
	(1.57, 3.48)	(0.916, 2.78)	(0.548, 2.39)
	p<0.001	p<0.001	p = 0.00179
round:early phase	**-0.319*****	**-0.197*****	**-0.152*****
	(-0.401, -0.238)	(-0.276, -0.117)	(-0.23, -0.0745)
	p<0.001	p<0.001	p<0.001
Random Effects			
group intercept sd	2.65	2.44	2.49

Table S16: Stability analysis of global actions. The results of CLMMs fit to global action counts in each of the a) Global Only, b) Balanced and c) Global Boost conditions.

	a. Balanced	b. Global Boost
	n(local)	n(local)
n(subgroups)	114	114
Fixed Effects		
round	0.00759	-0.0257
	(-0.0367, 0.0518)	(-0.0838, 0.0324)
	p = 0.737	p = 0.387
early phase	-0.505	0.184
	(-1.26, 0.251)	(-0.788, 1.16)
	p = 0.19	p = 0.71
round:early phase	0.0502	-0.0521
	(-0.0137, 0.114)	(-0.133, 0.029)
	p = 0.124	p = 0.208
Random Effects		
subgroup intercept sd	2.36	2.46

Table S17: Stability analysis of local actions. The results of CLMMs fit to action counts in each of the a) Balanced and b) Global Boost conditions.

	a. Global Only	b. Balanced	c. Global Boost
	n(defect)	n(defect)	n(defect)
n(groups)	57	57	57
Fixed Effects			
round	0.0346	0.0519	**0.0556*** **
	(-0.0205, 0.0898)	(-0.00116, 0.105)	(0.0454, 0.0658)
	p = 0.219	p = 0.0552	p<0.001
early phase	**-2.53*** **	**-1.71*** **	**-1.9*** **
	(-3.48, -1.57)	(-2.63, -0.797)	(-2.34, -1.46)
	p<0.001	p<0.001	p<0.001
round:early phase	**0.319*** **	**0.184*** **	**0.216*** **
	(0.238, 0.401)	(0.106, 0.262)	(0.158, 0.274)
	p<0.001	p<0.001	p<0.001
Random Effects			
Group intercept sd	2.65	1.39	2.01

Table S18: Stability analysis of defect actions. The results of CLMMs fit to action counts in each of the a) Global Only, b) Balanced and c) Global Boost conditions.

Behavioural Classification

In an exploratory analysis we classified participants based on their tendency to take particular actions. We classified participants as global-orientated, local-orientated, or free-riders if at least 67% of their choices matched that action; otherwise they were classified as ‘mixed’. We also classified participants as high best-responders if they chose a best response in at least 67% of their chosen actions (calculated ex-post from their group’s actions that round); otherwise they were classified as low best-responders. We chose 67% as it represents a clear majority of over two thirds of actions.

We report the distributions of classifications across experimental conditions and report chi-squared tests for the associations between i) proportion of action-orientated participants and experimental condition; ii) proportion of high best responders and experimental condition; iii) proportion of action orientation and proportion of high best-reponders across all conditions.

Figure S5 shows participant classifications by experimental condition. The proportion of players classified as action-orientated (classified into one of global-orientated, local-orientated, or free-riders) varied across conditions ($\chi^2(2) = 24.4$, $p < 0.001$; Global Only: 72.5%, Balanced: 54.7%, Global Boost: 66.1%). The proportion of players classified as best responders did not significantly vary across conditions ($\chi^2(2) = 5.10$, $p = 0.08$; Global Only: 15.5%, Balanced: 14.3%, Global Boost 9.9%), with an overall average across conditions of 13.2%. Action orientation was associated with best response classification ($\chi^2(3) = 296$, $p < 0.001$): across conditions; the proportion of high best responders was low among global-oriented (3.1%), low among mixed participants (6.0%), intermediate among local-orientated (35.9%) participants, and high among free-riders (54.6%).

Overall, these results are consistent with difficulties in coordinating and predicting other player's actions when working towards global cooperation, less difficulty in local cooperation, and irrelevance for free-riders.

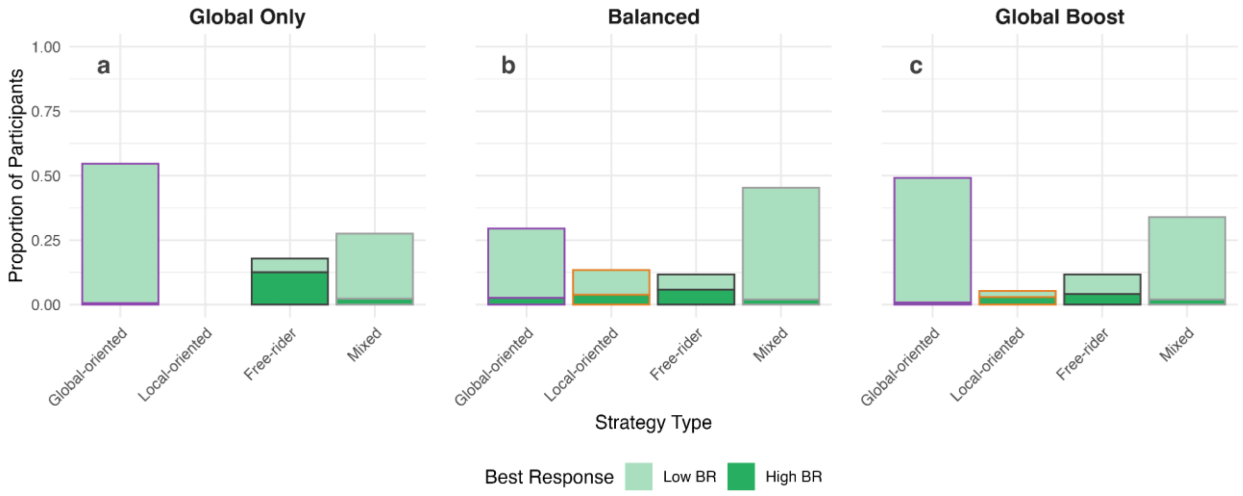

Figure S5: Participant classifications. Proportion of participants (y-axis) classified as global-oriented, local-oriented, free-rider, or mixed (at least 67% of choices in one action to be action-oriented, otherwise mixed). Dark green indicates high best responders (at least 67% best responses based on same-round group actions). a) Global Only: most players were global-orientated, with many mixed. b) Balanced: most players were mixed. c) Global Boost: most players were mixed, with many global-oriented. Across conditions there were correlations between action orientation and best responder classification: free-riders had the highest proportion of best responders, followed by local-orientated, then mixed, then global-orientated.

Successful Strategies

In an exploratory analysis we considered intra-group cooperation patterns to determine the strategies employed by successful groups. In particular, we were interested in whether stable cooperation was due to a core group of contributors or a more distributed pattern of participants sharing the duties of contributions across the group.

We examined the last 10 rounds of the game, where behavior is more likely to be stabilised than in the first 10 rounds. We selected globally successful groups as those who met the global cooperation threshold in at least 8 out of 10 of those rounds. For each successful group, we calculated the mean Jaccard similarity between the sets of global cooperators from one round to the next (intersection of sets of cooperators / union of sets of cooperators), which provides a measure of contributor consistency across rounds. We carried out the same analysis for local cooperation in subgroups. For reference, a Jaccard similarity of 1 indicates the same set of contributors each round; a value of 0.33 would indicate maximum rotation of the cooperating set between rounds (in both global and local cases).

We found 29 globally successful groups in the Global Only condition, 17 in the Balanced condition, and 26 in the Global Boost condition. These successful groups had high round-to-round stability in the set of global contributors (Jaccard similarity: 0.81 in Global Only, 0.77 in Balanced, 0.83 in Global Boost). Local cooperation showed a similar pattern (Balanced: 23 successful subgroups, Jaccard = 0.77; Global Boost: 8 successful subgroups; Jaccard = 0.84). In terms of both global and local successful groups, these results suggest that cooperation was achieved mainly through a stable core group of contributors with only occasional rotation.

References

1. Chen, D. L., Schonger, M. & Wickens, C. oTree—An open-source platform for laboratory, online, and field experiments. *Journal of Behavioral and Experimental Finance* **9**, 88–97 (2016).
2. Corazzini, L., Cotton, C. & Valbonesi, P. Donor coordination in project funding: Evidence from a threshold public goods experiment. *Journal of Public Economics* **128**, 16–29 (2015).
3. Croson, R. T. A. & Marks, M. B. Step Returns in Threshold Public Goods: A Meta- and Experimental Analysis. *Experimental Economics* **2**, 239–259 (2000).
4. Fischbacher, U. & Gächter, S. Social preferences, beliefs, and the dynamics of free riding in public goods experiments. *American economic review* **100**, 541–556 (2010).
5. Rapoport, A. Provision of step-level public goods: Effects of inequality in resources. *Journal of Personality and Social Psychology* **54**, 432–440 (1988).

6. Balliet, D., Wu, J. & De Dreu, C. K. W. Ingroup favoritism in cooperation: A meta-analysis. *Psychological Bulletin* **140**, 1556–1581 (2014).
7. Romano, A., Balliet, D., Yamagishi, T. & Liu, J. H. Parochial trust and cooperation across 17 societies. *Proceedings of the National Academy of Sciences* **114**, 12702–12707 (2017).
8. Romano, A., Sutter, M., Liu, J. H., Yamagishi, T. & Balliet, D. National parochialism is ubiquitous across 42 nations around the world. *Nature communications* **12**, 4456 (2021).
9. Buchan, N. R. *et al.* Globalization and human cooperation. *Proceedings of the National Academy of Sciences* **106**, 4138–4142 (2009).
10. Böhm, R., Bornstein, G. & Koppel, H. *Between-Group Conflict and Other-Regarding Preferences in Nested Social Dilemmas*. (2014).
11. Brewer, M. B., Buchan, N. R., Ozturk, O. D. & Grimalda, G. Parochial Altruism and Political Ideology. *Political Psychology* **44**, 383–396 (2023).
12. Waytz, A., Iyer, R., Young, L., Haidt, J. & Graham, J. Ideological differences in the expanse of the moral circle. *Nat Commun* **10**, 4389 (2019).
13. Buchan, N. R. *et al.* Global Social Identity and Global Cooperation. *Psychol Sci* **22**, 821–828 (2011).
14. Chakravarty, S. & Fonseca, M. A. Discrimination via Exclusion: An Experiment on Group Identity and Club Goods. *Journal of Public Economic Theory* **19**, 244–263 (2017).
15. Gallier, C. *et al.* Leveling up? An inter-neighborhood experiment on parochialism and the efficiency of multi-level public goods provision. *Journal of Economic Behavior & Organization* **164**, 500–517 (2019).
16. Wit, A. P. & Kerr, N. L. ‘Me versus just us versus us all’ categorization and cooperation in nested social dilemmas. *Journal of Personality and Social Psychology* **83**, 616–637 (2002).
17. Beal, D. J., Cohen, R. R., Burke, M. J. & McLendon, C. L. Cohesion and performance in groups: a meta-analytic clarification of construct relations. *Journal of applied psychology* **88**, 989 (2003).

18. Mann, H. B. & Whitney, D. R. On a test of whether one of two random variables is stochastically larger than the other. *The annals of mathematical statistics* 50–60 (1947).
19. Team, R. C. R: A language and environment for statistical computing. *Foundation for Statistical Computing, Vienna, Austria* (2013).
20. Holm, S. A simple sequentially rejective multiple test procedure. *Scandinavian journal of statistics* 65–70 (1979).
21. Christensen, R. H. B. Cumulative link models for ordinal regression with the R package ordinal. *Submitted in J. Stat. Software* **35**, (2018).
22. Singmann, H., Bolker, B., Westfall, J., Aust, F. & Ben-Shachar, M. S. afex: Analysis of factorial experiments. *R package version 0.13–145* (2015).
23. Gerpott, F. H., Balliet, D., Columbus, S., Molho, C. & de Vries, R. E. How do people think about interdependence? A multidimensional model of subjective outcome interdependence. *Journal of Personality and Social Psychology* **115**, 716–742 (2018).
